# Supplementary material for: LCMSpector: A simple open-source viewer for targeted hyphenated mass spectrometry analysis
Source: PLoS Comput Biol. 2025 Dec 15;21(12):e1013095. doi: 10.1371/journal.pcbi.1013095 (PMC12725707; doi:10.1371/journal.pcbi.1013095)
Supplement: S1 Text. — Fig A. (A) The MVC architectural design of LCMSpector, created in BioRender with a publication license. Wetter, E. (2025) https://BioRender.com/4e4i8uvand. (B) LCMSpector data flow diagram. Fig B. Roadmap showing the plans for future development of LCMSpector. Table A. Windows—results of performance benchmarking between LCMSpector, MZMine and Sirius loading the same sets of mzML files. Table B. MacOS—results of performance benchmarking between LCMSpector, MZMine and Sirius loading the same sets of mzML files. Table C. Linux—results of performance benchmarking between LCMSpector, MZMine and Sirius loading the same sets of mzML files. Fig D. Calibration curve of aspartic acid from the calibration panel in Chromeleon Chromatography Studio (ver. 7.3.2) and calibration curve created based on the integrated intensities created in R (log–log). Fig E. Calibration curve of glutamic acid from the calibration panel in Chromeleon Chromatography Studio (ver. 7.3.2) and calibration curve created based on the integrated intensities created in R (log–log). Fig F. Calibration curve of histidine from the calibration panel in Chromeleon Chromatography Studio (ver. 7.3.2) and calibration curve created based on the integrated intensities created in R (log–log). Fig G. Calibration curve of lysine from the calibration panel in Chromeleon Chromatography Studio (ver. 7.3.2) and calibration curve created based on the integrated intensities created in R (log–log). Fig H. Calibration curve of serine from the calibration panel in Chromeleon Chromatography Studio (ver. 7.3.2) and calibration curve created based on the integrated intensities created in R (log–log). Fig I Calibration curve (log–log) of aspartic acid produced in R based on the integrated peak areas from MZmine version 4.7.28. Fig J. Calibration curve (log–log) of glutamic acid produced in R based on the integrated peak areas from MZmine version 4.7.28. Fig K. Calibration curve (log–log) of histidine produced in R based on the integ [file pcbi.1013095.s001.docx]

# Supporting Information for the manuscript “LCMSpector: a simple open-source viewer for targeted hyphenated mass spectrometry analysis”

Table of Contents

[Supporting Information for the manuscript “LCMSpector: a simple open-source viewer for targeted hyphenated mass spectrometry analysis” 1](#_Toc215949925)

[Application design and future directions 2](#_Toc215949926)

[Performance benchmarking 4](#_Toc215949927)

[**Table A.** Windows – results of performance benchmarking between LCMSpector, MZMine and Sirius loading the same sets of mzML files. 4](#_Toc215949928)

[**Table B.** MacOS – results of performance benchmarking between LCMSpector, MZMine and Sirius loading the same sets of mzML files. 5](#_Toc215949929)

[**Table C.** Linux – results of performance benchmarking between LCMSpector, MZMine and Sirius loading the same sets of mzML files. 6](#_Toc215949930)

[Calibration curves 7](#_Toc215949931)

[Chromeleon 7.3.2 7](#_Toc215949932)

[8](#_Toc215949933)

[MZMine 4.7.29 12](#_Toc215949934)

[LCMSpector 0.9.10 17](#_Toc215949935)

[Appendix A 22](#_Toc215949936)

[Instructions for the usability study of LCMSpector 22](#_Toc215949937)

[Appendix B 27](#_Toc215949938)

[Contents of the usability study questionnaire 27](#_Toc215949939)

## Application design and future directions


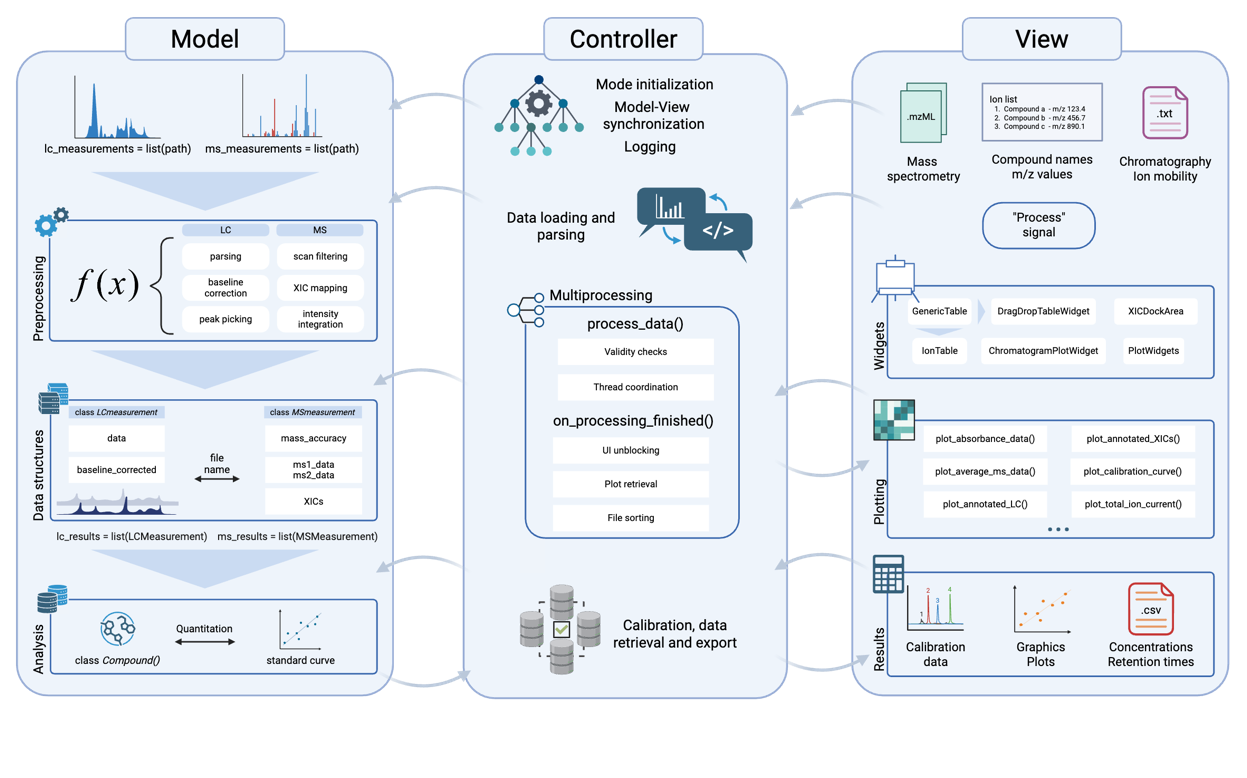


**A**


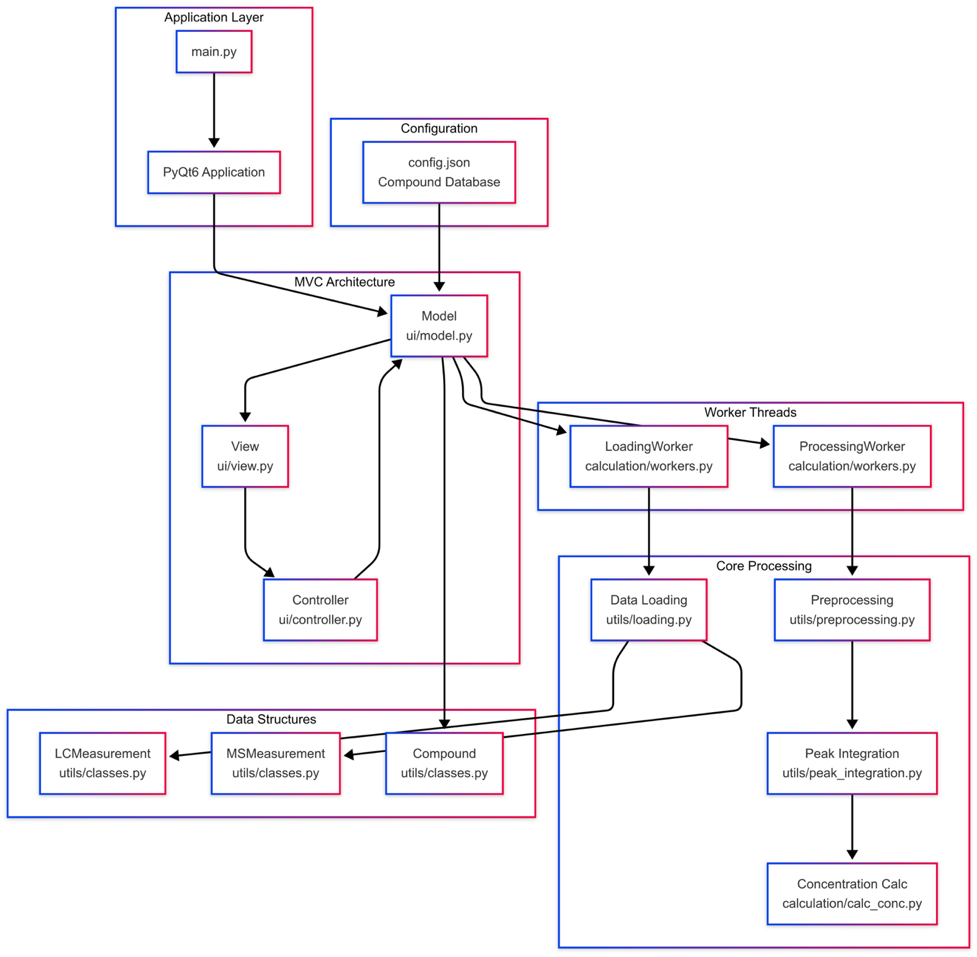


**B**

**Fig. A. (A)** The MVC architectural design of LCMSpector, created in BioRender. Wetter, E. (2025) https://BioRender.com/4e4i8uvand. **(B)** LCMSpector data flow diagram.


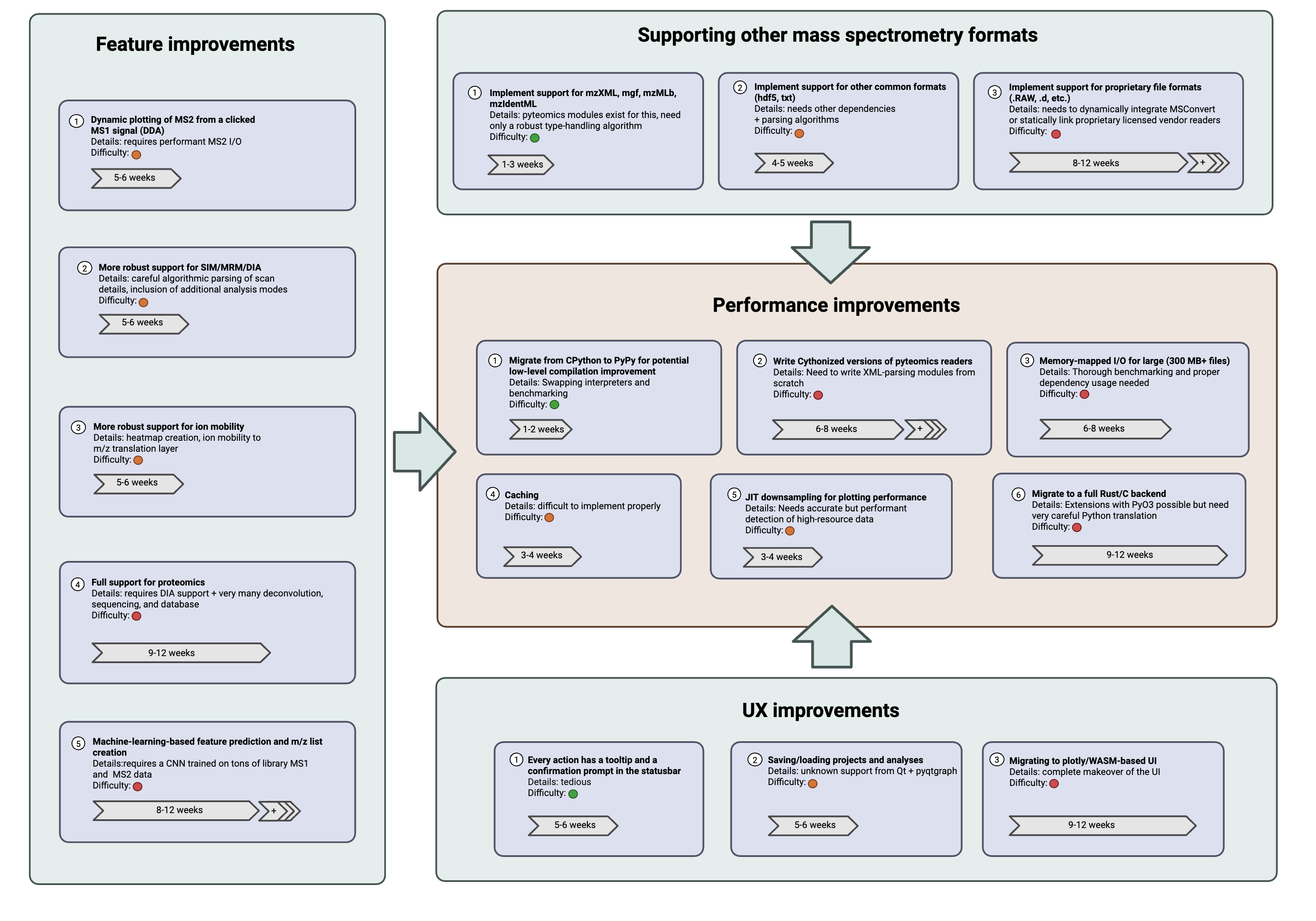


**Fig. B**. Roadmap showing the plans for future development of LCMSpector.

## Performance benchmarking

### **Table A.** Windows – results of performance benchmarking between LCMSpector, MZMine and Sirius loading the same sets of mzML files.

| Windows 10 IoT Enterprise LTSC | 12th Gen Intel(R) Core(TM) i7-12700, 2.10 GHz  32 GB RAM  12 cores | | | | | | | | | |
| --- | --- | --- | --- | --- | --- | --- | --- | --- | --- | --- |
|  | MZMine 4.7.29 | | | LCMSpector 0.9.10 | | | OpenMS TOPPView 3.4.1 | | | Total size on disk (GB) |
|  | Loading time (s) | Processing time (s) | Peak RAM (GB) | Loading time (s) | Processing time (s) | Peak RAM (GB) | Loading time (s) | Processing time (s) | Peak RAM (GB) |  |
| baseline (application only) | 0 | 0 | 0.49 | 0 | 0 | 0.25 | 0 | N/A | 0.03 | - |
| 8 files | 1 | 1 | 2 | 4.03 | 2.52 | 0.62 | 11.58 | N/A | 0.6 | 0.31 |
| 16 files | 1 | 1 | 2.58 | 5.2 | 3.99 | 0.91 | 15.73 | N/A | 1.2 | 0.6 |
| 32 files | 2 | 2 | 4.01 | 7.61 | 5.23 | 1.5 | 30.35 | N/A | 2.41 | 1.15 |
| 64 files | 4 | 2 | 10.45 | 12.63 | 9.3 | 2.68 | 76.86 | N/A | 4.54 | 2.34 |
| 128 files | 11 | 2 | 15.05 | 21.94 | 15.67 | 4.52 | 108.72 | N/A | 8.25 | 4.51 |
| 256 files | 33 | 3 | 23.75 | 35.57 | 25.3 | 6.85 | 174.23 | N/A | 12.34 | 7.74 |

### **Table B.** MacOS – results of performance benchmarking between LCMSpector, MZMine and Sirius loading the same sets of mzML files.

| MacOS Sonoma 15.6.1 | Apple M3 Max  36 GB RAM  14 cores | | | | | | | | | |
| --- | --- | --- | --- | --- | --- | --- | --- | --- | --- | --- |
|  | MZMine 4.7.29 | | | LCMSpector 0.9.10 | | | OpenMS TOPPView 3.4.1 | | | Total size on disk (GB) |
|  | Loading time (s) | Processing time (s) | Peak RAM (GB) | Loading time (s) | Processing time (s) | Peak RAM (GB) | Loading time (s) | Processing time (s) | Peak RAM (GB) |  |
| baseline (application only) | 0 | 0 | 1.02 | 0 | 0 | 0.3 | 0 | N/A | 0.13 | - |
| 8 files | 1 | 1 | 2.42 | 1.9 | 1.12 | 0.36 | 2.98 | N/A | 0.57 | 0.31 |
| 16 files | 2 | 1 | 2.84 | 2.66 | 1.45 | 0.72 | 6.65 | N/A | 1.02 | 0.6 |
| 32 files | 3 | 1 | 4.56 | 4.01 | 2.36 | 1.29 | 12.55 | N/A | 1.8 | 1.15 |
| 64 files | 4 | 1 | 7.93 | 7.5 | 3.55 | 2.27 | 34.57 | N/A | 4.26 | 2.34 |
| 128 files | 7 | 2 | 11.42 | 13.37 | 6.71 | 4.44 | 73.31 | N/A | 7.95 | 4.51 |
| 256 files | 10 | 10 | 14.26 | 20.75 | 12.35 | 7.45 | 159.21 | N/A | 12.9 | 7.74 |

### **Table C.** Linux – results of performance benchmarking between LCMSpector, MZMine and Sirius loading the same sets of mzML files.

| Fedora Linux 42 | AMD Ryzen 7 7800X3D @ 5.05 GHz  64 GB RAM  16 cores | | | | | | | | | |
| --- | --- | --- | --- | --- | --- | --- | --- | --- | --- | --- |
|  | MZMine 4.7.29 | | | LCMSpector 0.9.10 | | | OpenMS TOPPView 3.4.1 | | | Total size on disk (GB) |
|  | Loading time (s) | Processing time (s) | Peak RAM (GB) | Loading time (s) | Processing time (s) | Peak RAM (GB) | Loading time (s) | Processing time (s) | Peak RAM (GB) |  |
| baseline (application only) | 0 | 0 | 0.53 | 0.00 | 0.00 | 0.46 | 0 | N/A | 0.11 | - |
| 8 files | 1 | 1 | 3.7 | 1.67 | 0.93 | 0.91 | 3.75 | N/A | 0.69 | 0.31 |
| 16 files | 1 | 1 | 4 | 3.30 | 1.58 | 1.10 | 7.33 | N/A | 1.2 | 0.6 |
| 32 files | 2 | 1 | 5.2 | 4.93 | 3.15 | 1.90 | 14.31 | N/A | 2 | 1.15 |
| 64 files | 3 | 1 | 10.7 | 11.36 | 4.65 | 2.70 | 37.44 | N/A | 5.5 | 2.34 |
| 128 files | 4 | 2 | 16.1 | 19.00 | 8.88 | 4.60 | 73.31 | N/A | 8.8 | 4.51 |
| 256 files | 6 | 2 | 28.3 | 29.99 | 14.96 | 7.00 | 150.69 | N/A | 13 | 7.74 |

## Calibration curves

### Chromeleon 7.3.2

Summary of the calibration curves generated by Chromeleon (linear regression, top figure, unused) and recalculated log-log plots generated in R from the integrated intensities obtained from Chromeleon.


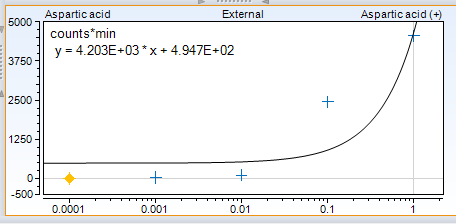

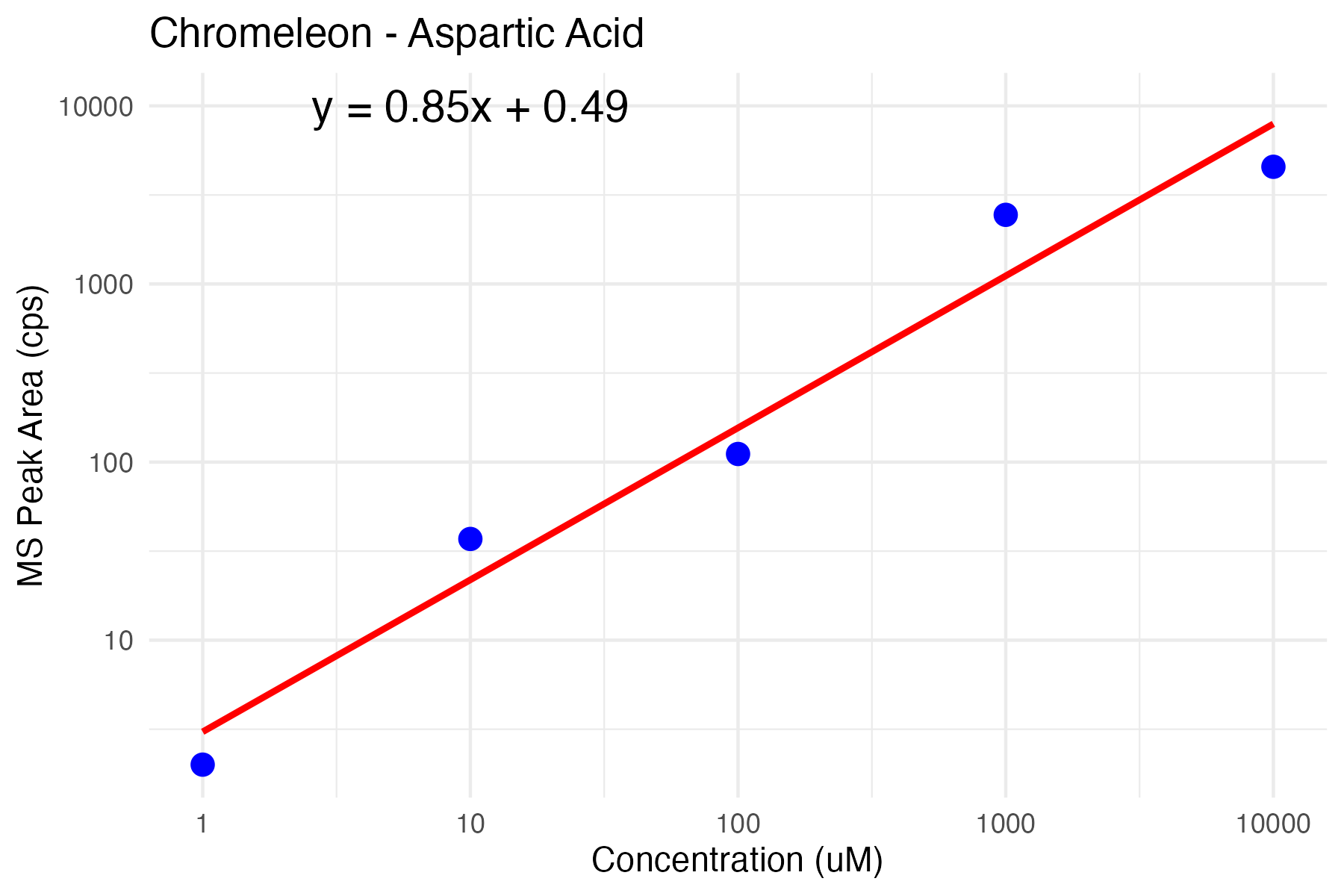


**Fig. D.** Calibration curve of aspartic acid from the calibration panel in Chromeleon Chromatography Studio (ver. 7.3.2) and calibration curve created based on the integrated intensities created in R (log-scale).

###
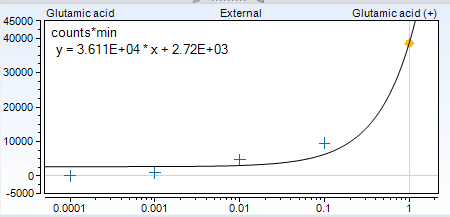


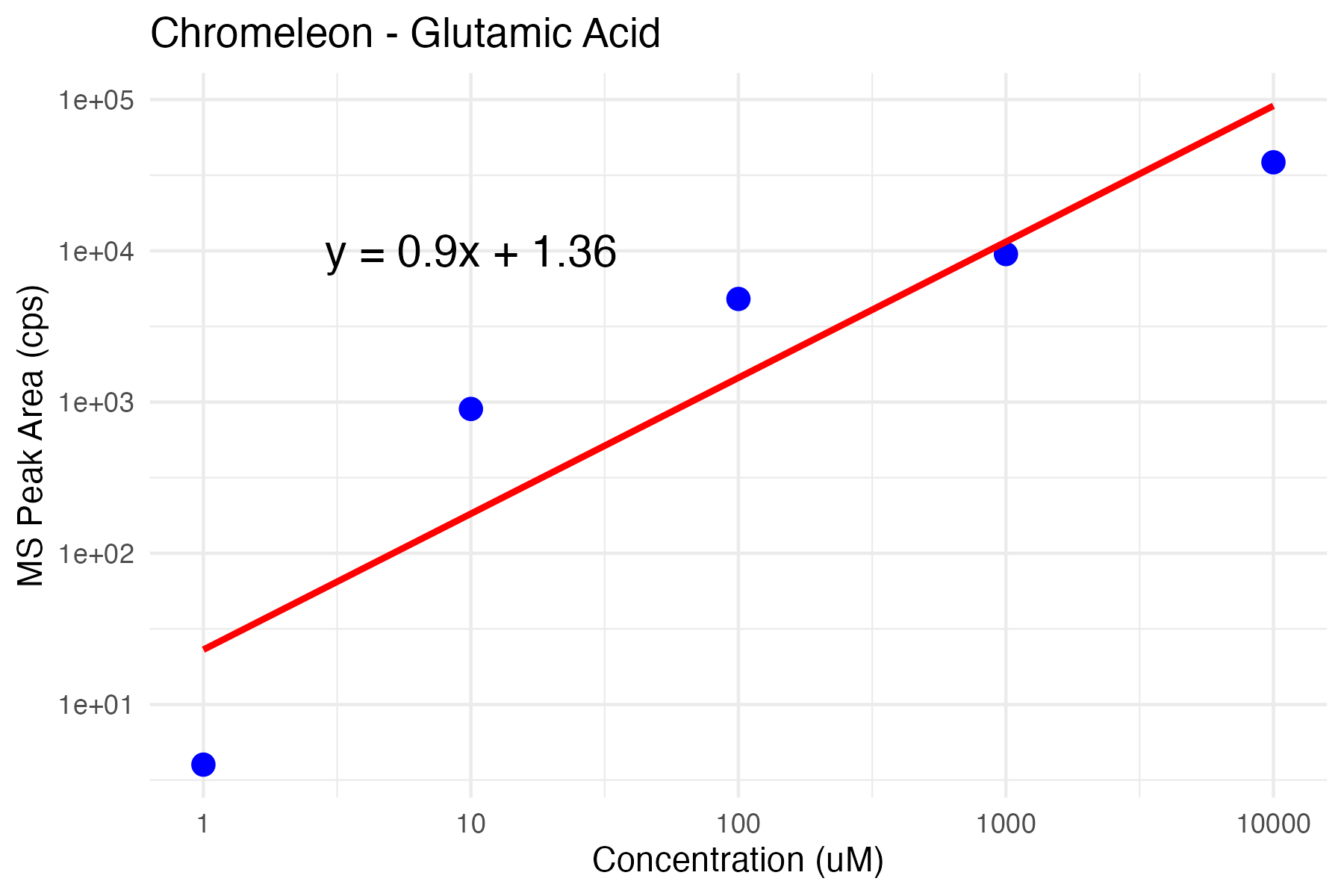


**Fig. E.** Calibration curve of glutamic acid from the calibration panel in Chromeleon Chromatography Studio (ver. 7.3.2) and calibration curve created based on the integrated intensities created in R (log-scale).


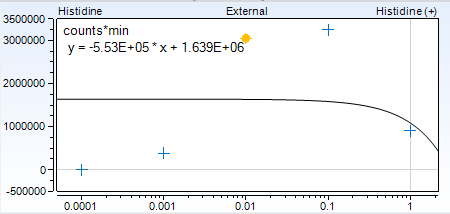


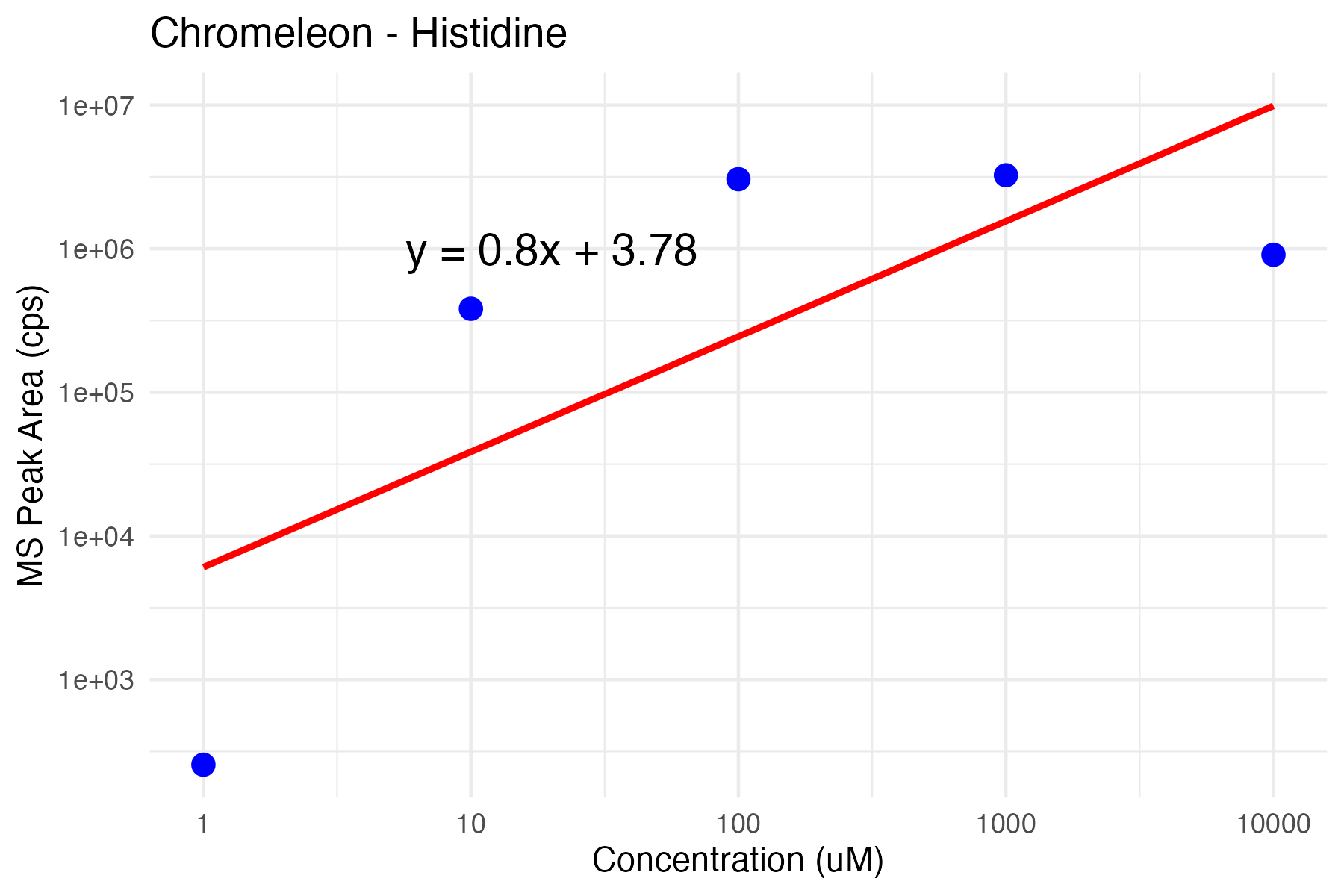


**Fig. F.** Calibration curve of histidine from the calibration panel in Chromeleon Chromatography Studio (ver. 7.3.2) and calibration curve created based on the integrated intensities created in R (log-scale).


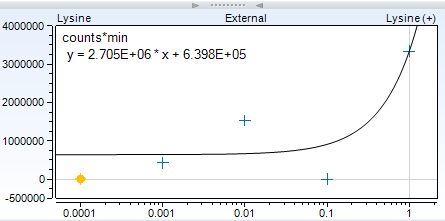


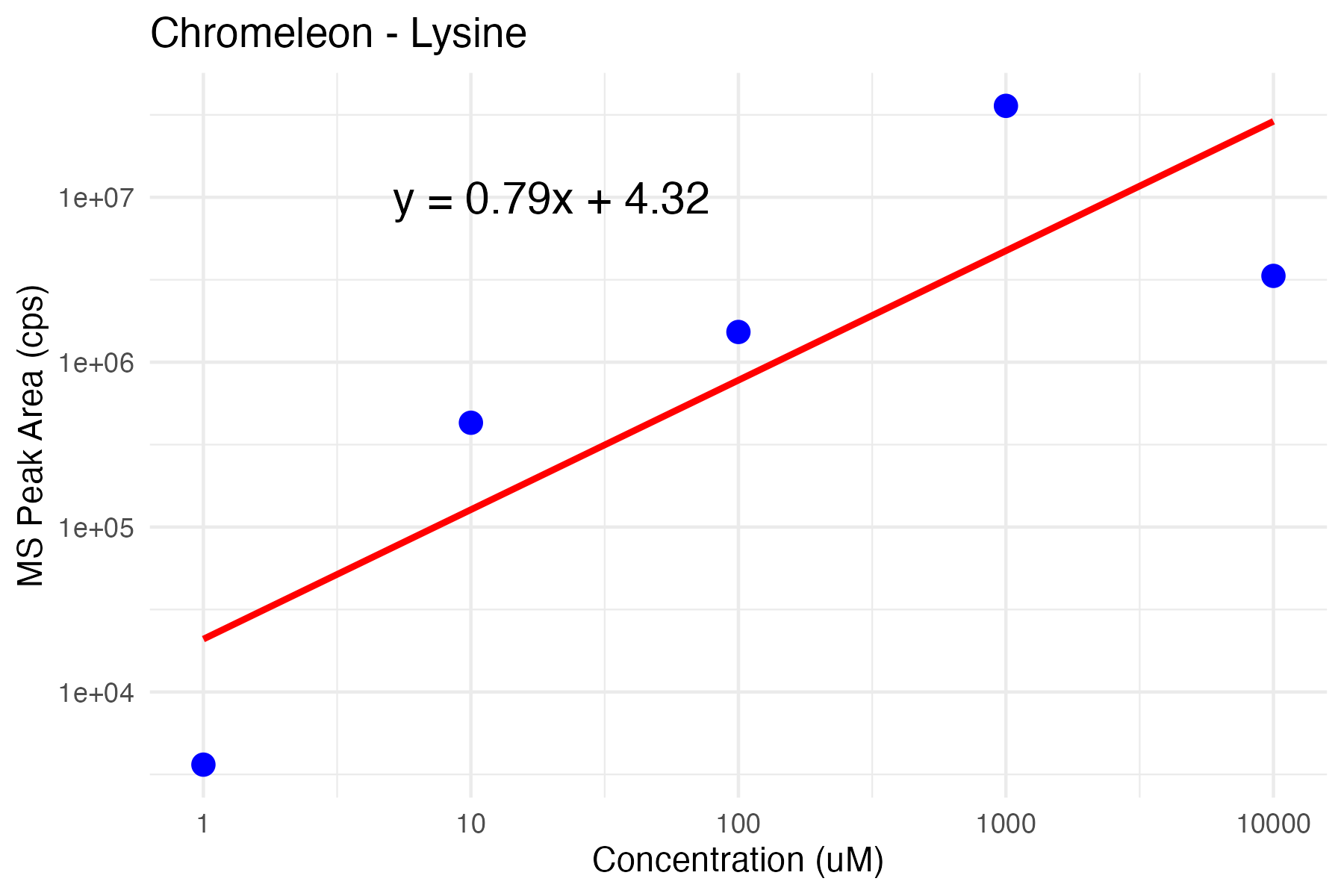


**Fig. G.** Calibration curve of lysine from the calibration panel in Chromeleon Chromatography Studio (ver. 7.3.2) and calibration curve created based on the integrated intensities created in R (log-scale).


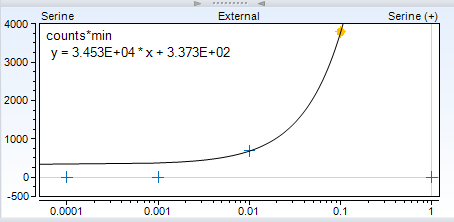


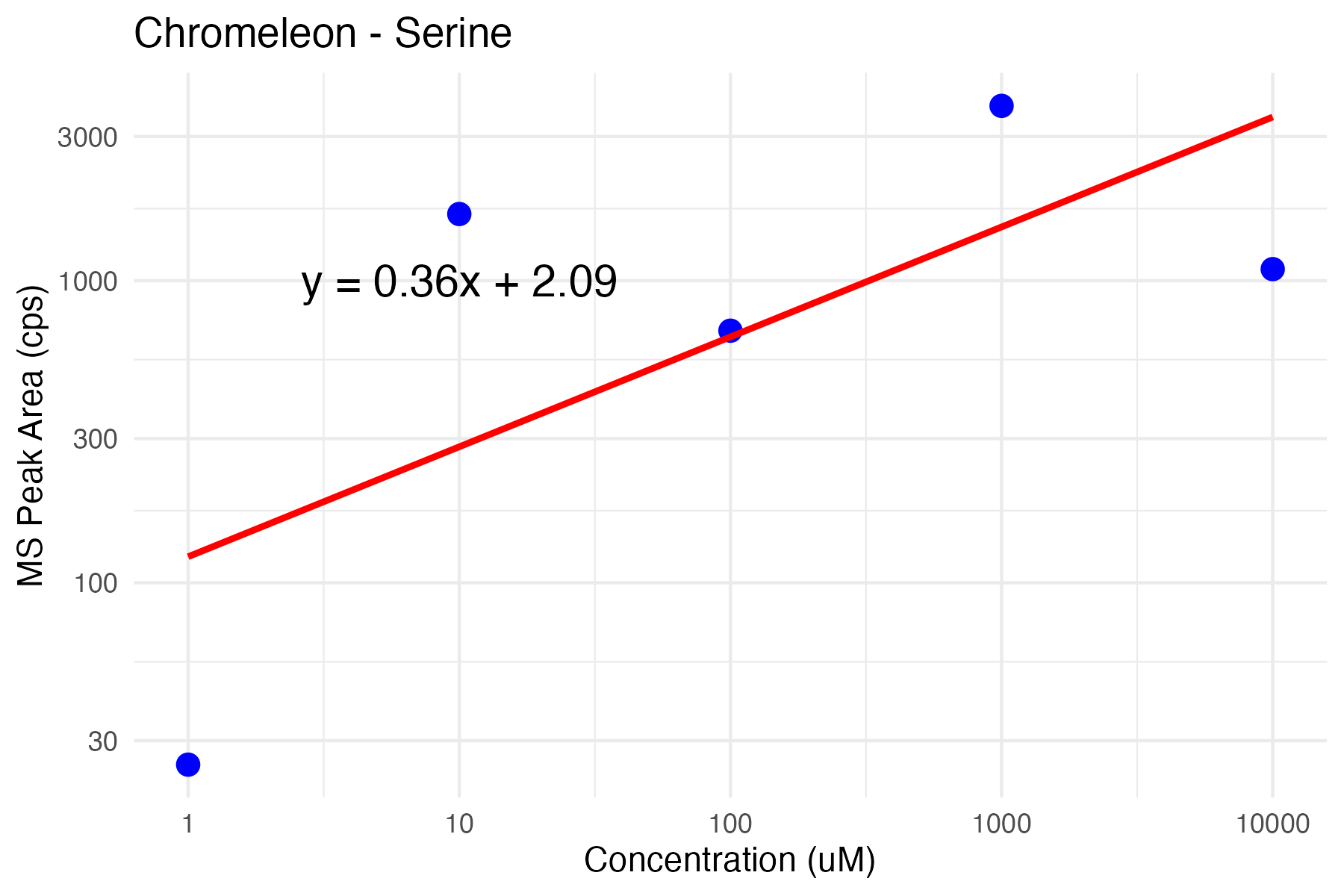


**Fig. H.** Calibration curve of serine from the calibration panel in Chromeleon Chromatography Studio (ver. 7.3.2) and calibration curve created based on the integrated intensities created in R (log-scale).

### MZMine 4.7.29

**
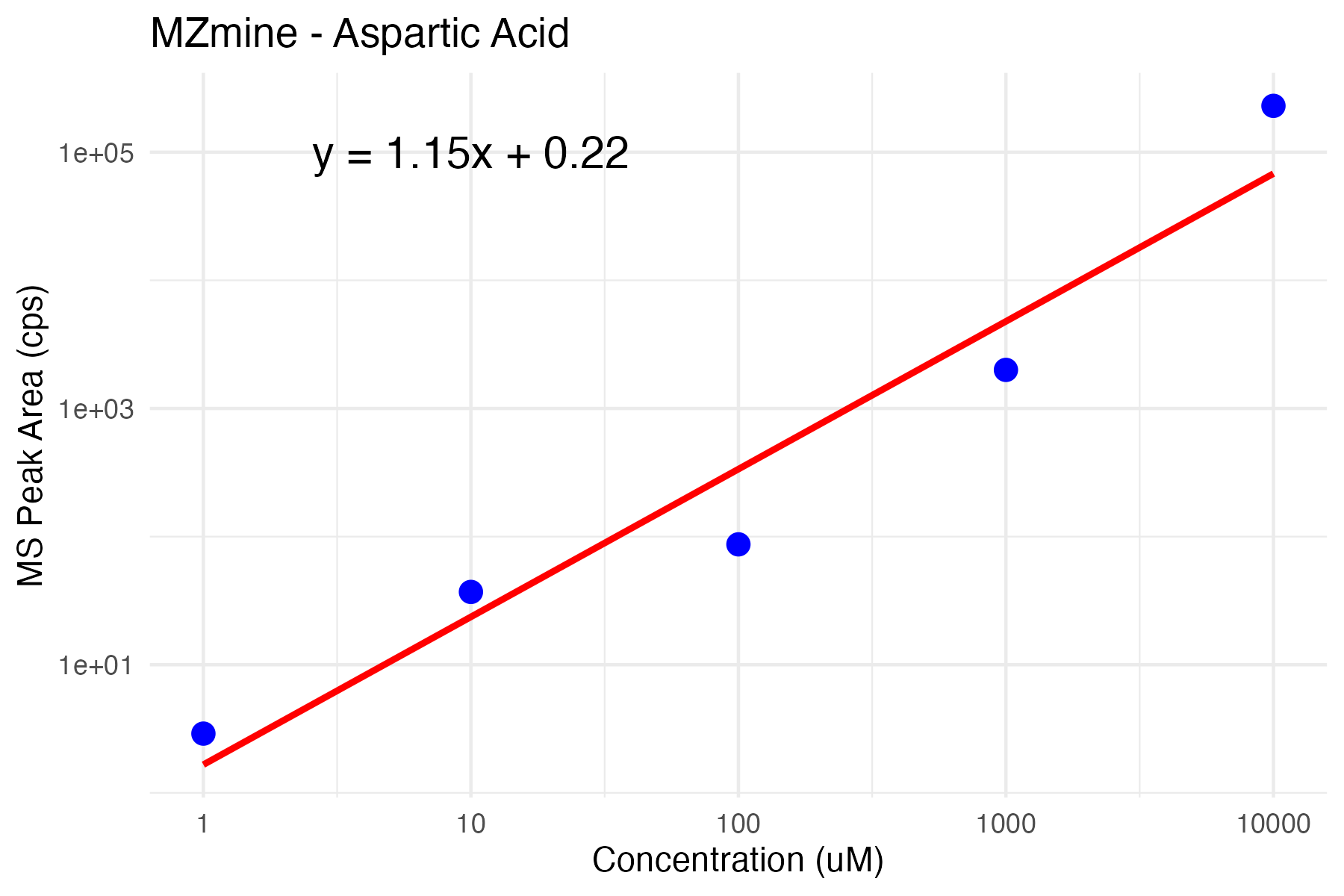
**

**Fig. I.** Calibration curve (log-log) of aspartic acid produced in R based on the integrated peak areas from MZmine version 4.7.28


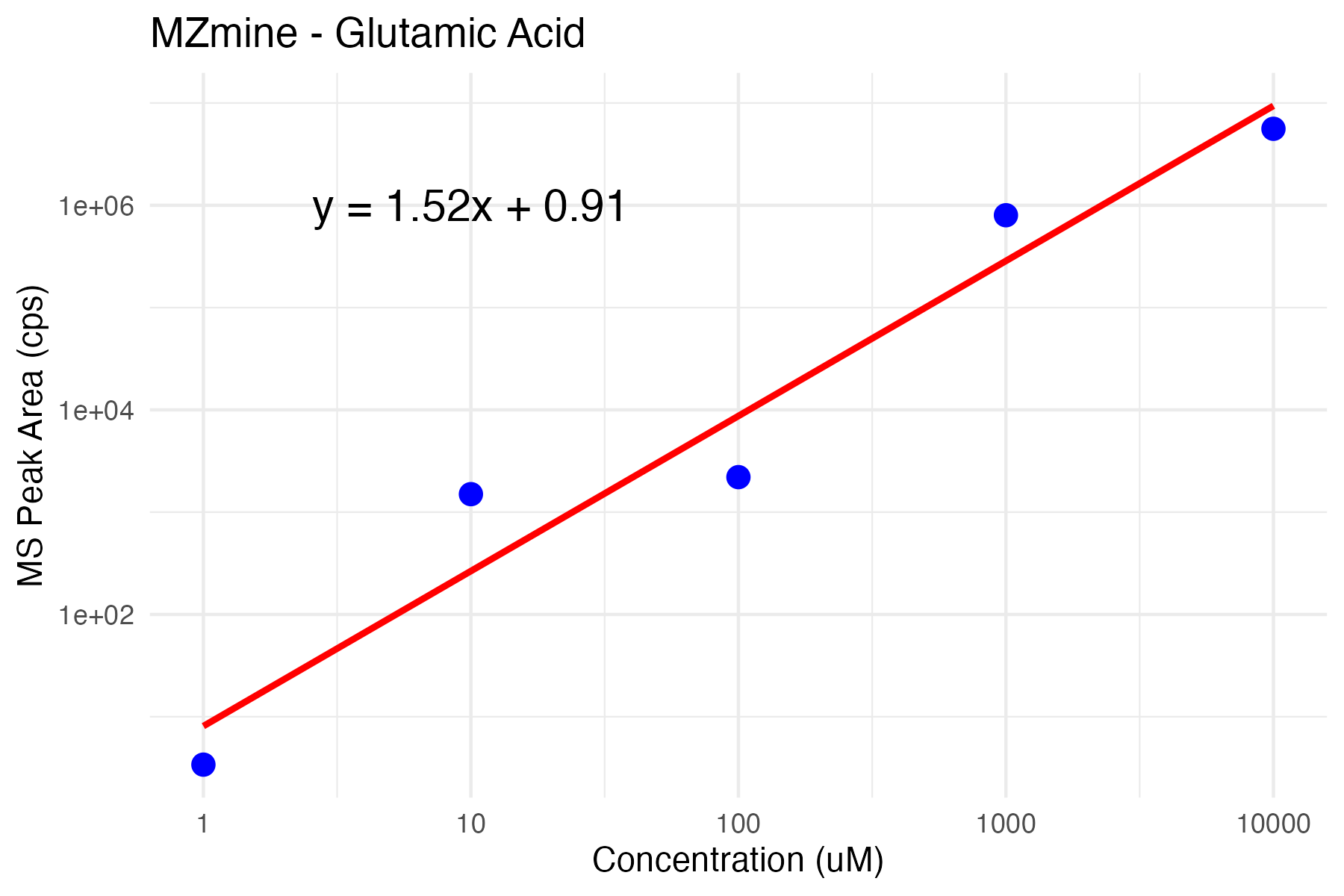


**Fig. J.** Calibration curve (log-log) of glutamic acid produced in R based on the integrated peak areas from MZmine version 4.7.28.


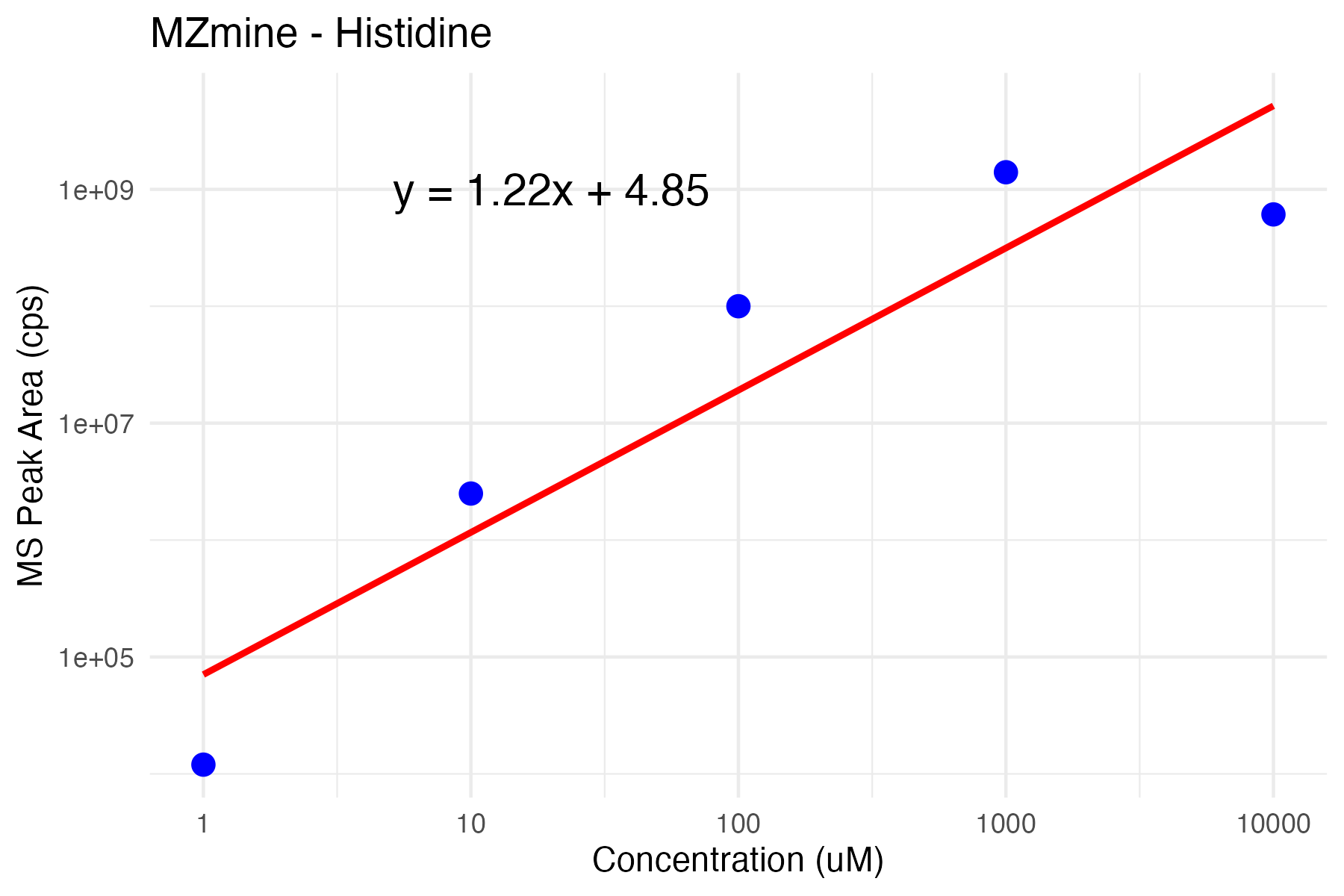


**Fig. K.** Calibration curve (log-log) of histidine produced in R based on the integrated peak areas from MZmine version 4.7.28.


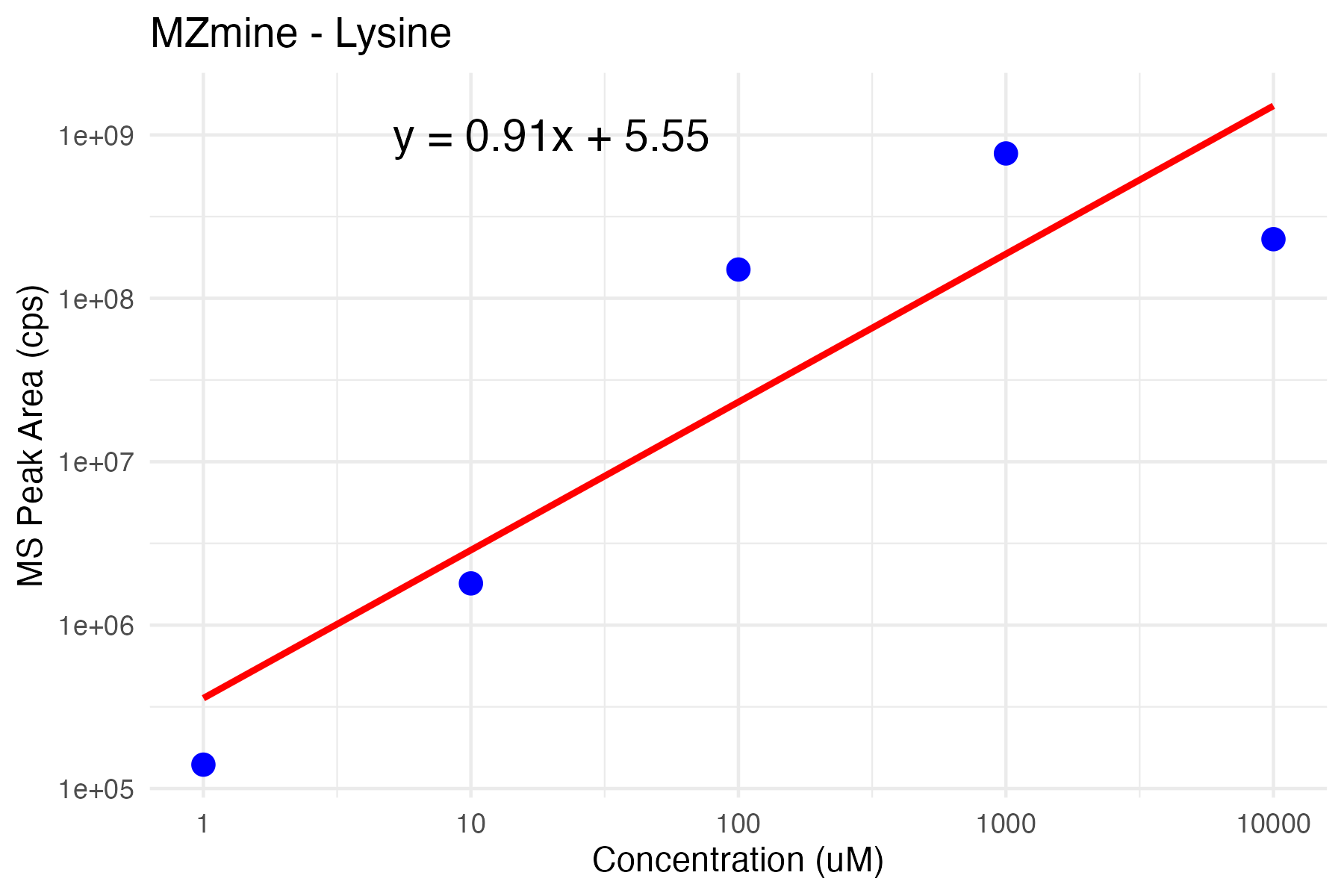


**Fig. L.** Calibration curve (log-log) of lysine produced in R based on the integrated peak areas from MZmine version 4.7.28.


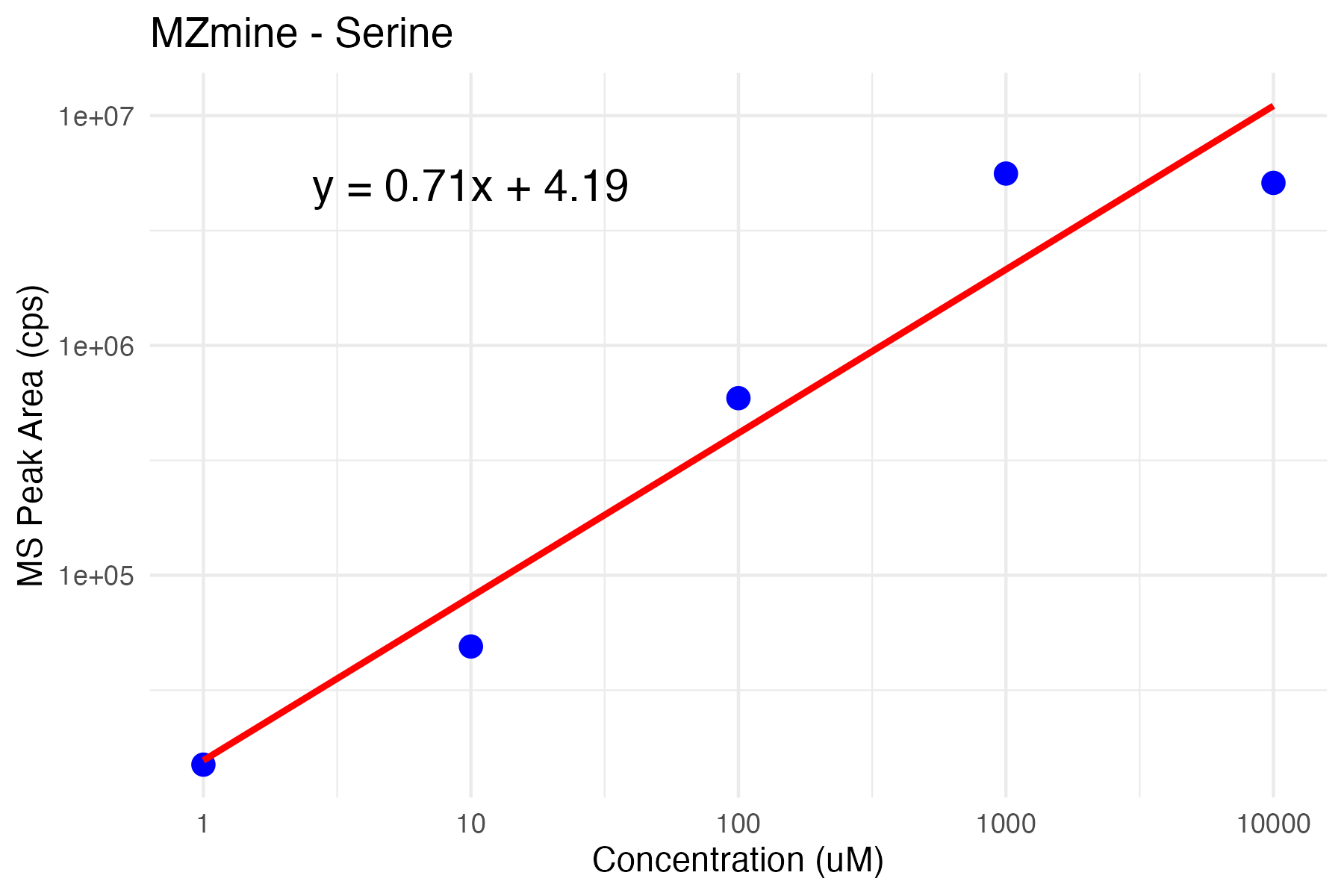


**Fig. M.** Calibration curve (log-log) of serine produced in R based on the integrated peak areas from MZmine version 4.7.28.

### LCMSpector 0.9.10


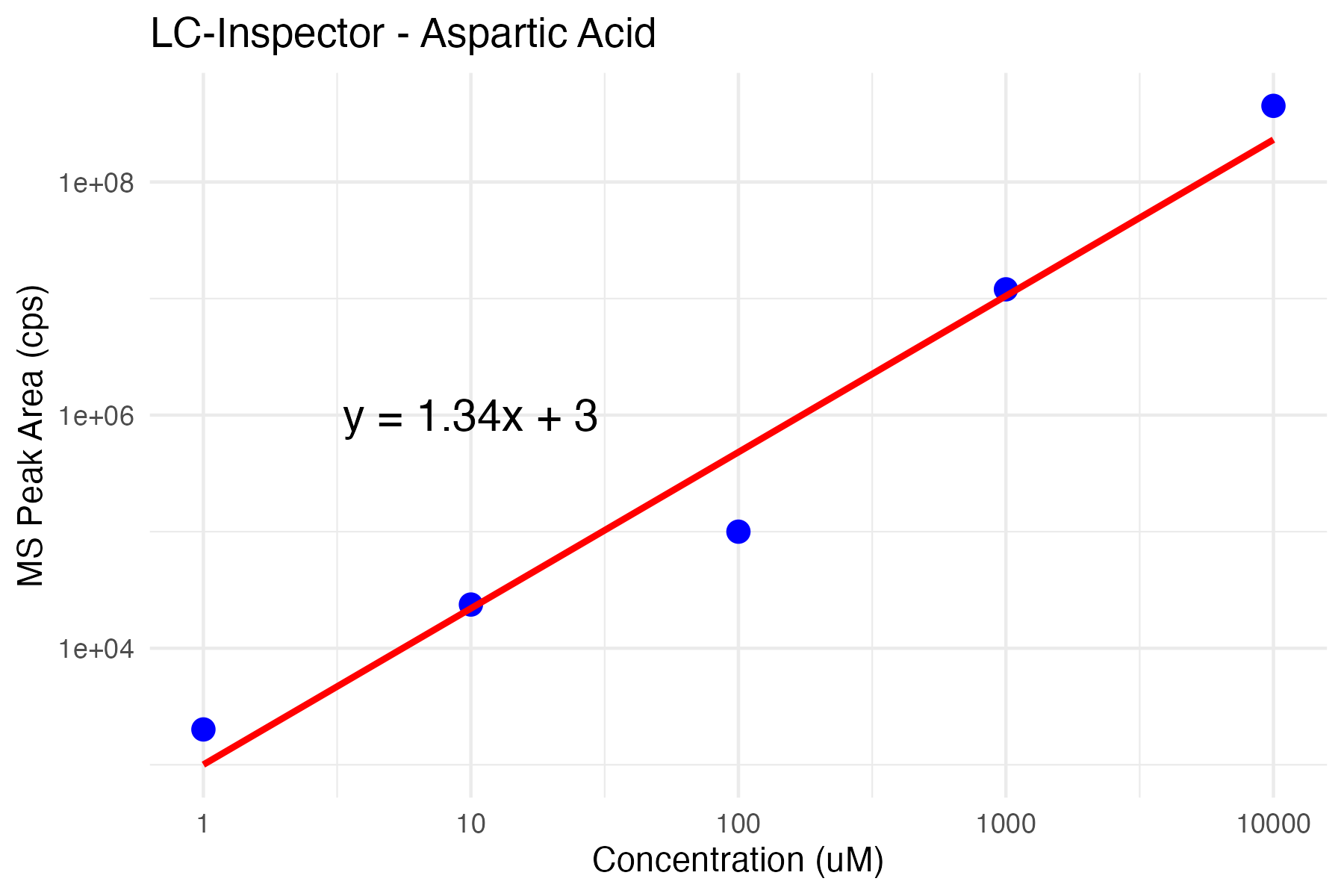


**Fig. N.** Calibration curve (log-log) of aspartic acid produced in R based on the integrated peak areas from LCMSpector 0.9.10.


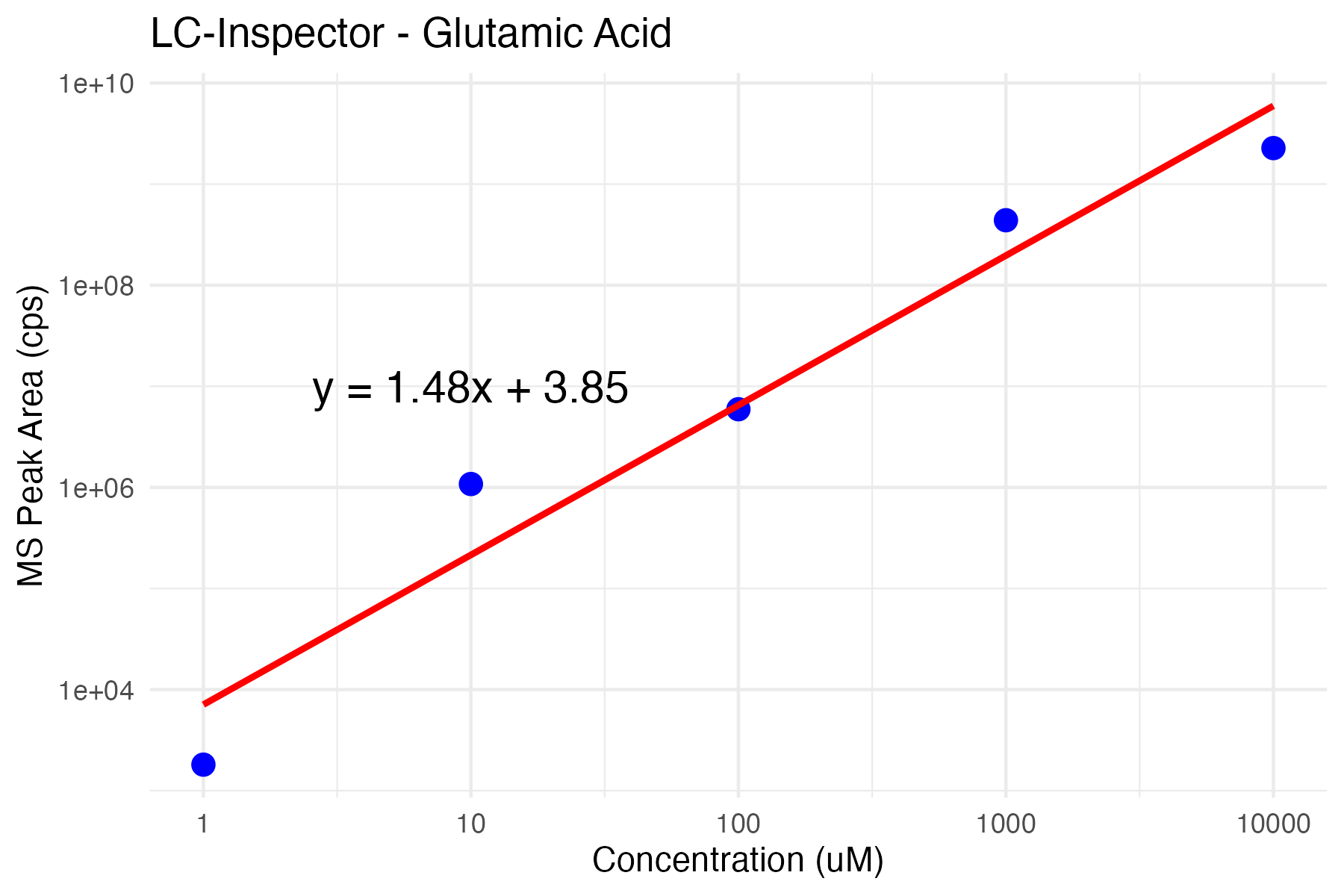


**Fig. O.** Calibration curve of glutamic acid produced in R based on the integrated peak areas from LCMSpector 0.9.10.


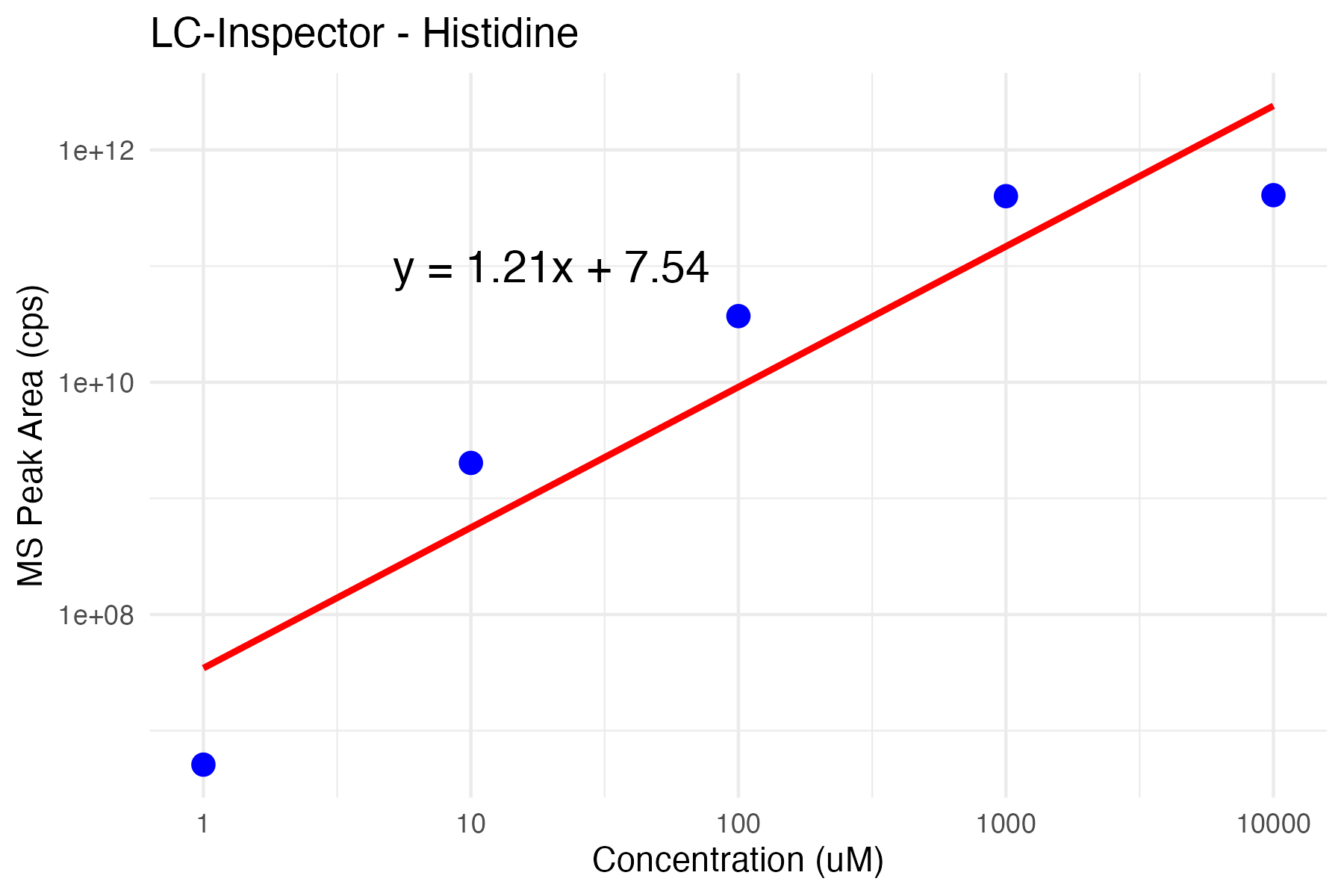


**Fig. P.** Calibration curve (log-log) of histidine produced in R based on the integrated peak areas from LCMSpector 0.9.10.


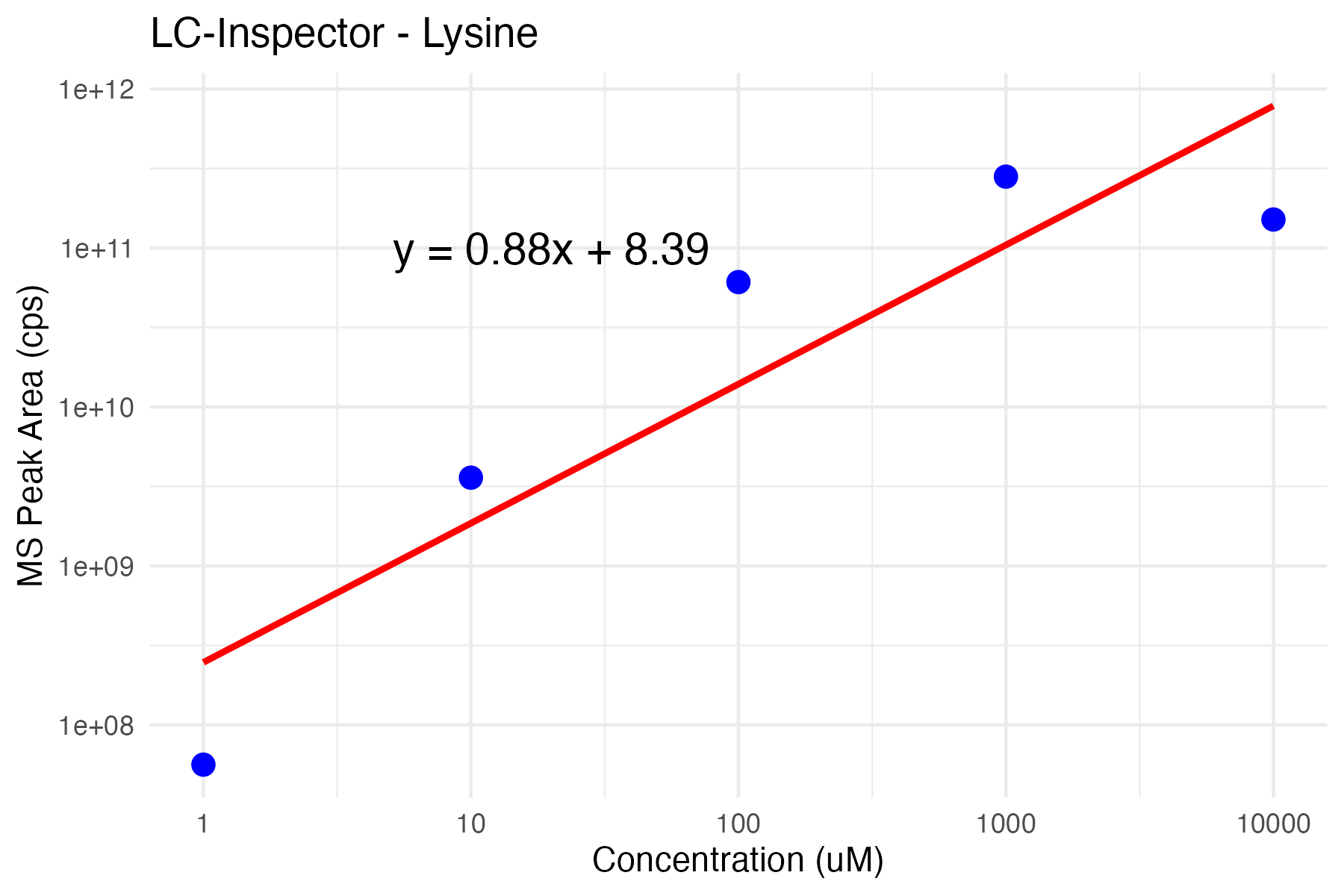


**Fig. Q.** Calibration curve (log-log) of lysine produced in R based on the integrated peak areas from LCMSpector 0.9.10.


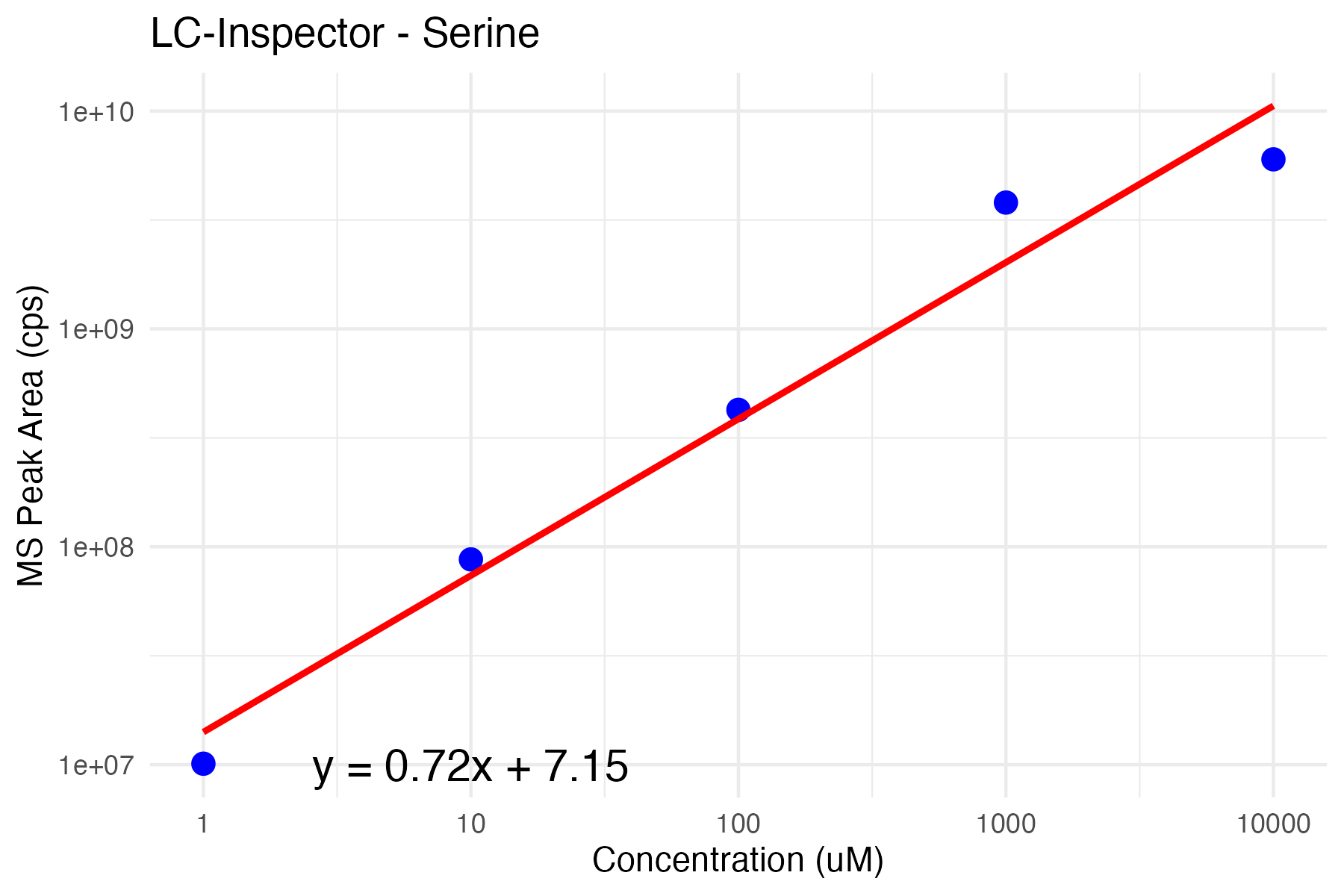


**Fig. R.** Calibration curve (log-log) of serine produced in R based on the integrated peak areas from LCMSpector 0.9.10.

# Appendix A

## Instructions for the usability study of LCMSpector

**LCMSpector – Step-by-Step Beginner’s Guide**

*(No mass spectrometry knowledge required)*

**Welcome**

Thank you for helping us test LCMSpector! This guide will walk you through:

1. Installing LCMSpector on your computer
2. Downloading and opening a sample dataset
3. Loading that sample into LCMSpector
4. Choosing an ion/metabolite list to search for
5. Running the analysis
6. Looking at the results
7. Exporting the results

You can follow along **even if you have never used scientific software before.**

**Part 1: Installing LCMSpector**

**Step 1 – Download LCMSpector**

1. Open this page in your browser:

[**LCMSpector Releases on GitHub**](https://github.com/MateuszFido/LC-Inspector/releases)

1. Scroll to the **latest release** at the top.
2. Under “Assets”, download the right version for your computer:
   - **Windows:** Click the .zip file containing “Win11” in the name
   - **macOS:** Click the .zip file containing “macOS” in the name
   - **Linux:** Click the .zip file containing “Linux” in the name

**Step 2 – Open and Install**

**If you are on Windows**

1. Unpack the archive, go into LC-Inspector/dist and find the .exe file.
2. Double-click the .exe file.
3. **You might see a blue “Windows protected your PC” screen**.
   - Click **More info**.
   - Click **Run anyway**.

*(This happens because the app is not from the Microsoft Store — it does not mean it is dangerous).*

1. If you see a “Windows Defender SmartScreen” pop-up in *Protection history*, open it, select LCMSpector, and click **Restore**.

**If you are on macOS**

1. Double-click the downloaded file to open.
2. If you see a message saying LCMSpector **“cannot be opened because it is from an unidentified developer”**:
   - Go to **System Settings → Privacy & Security**
   - Scroll down and click **Open Anyway** next to LCMSpector.
3. If the system tells you the file is “quarantined” or “broken and should be moved to the Trash”:
   - Click “Done”, not “Move to Trash” • Open **Terminal** (in Applications → Utilities)
   - Type:

*xattr -d com.apple.quarantine /path/to/LCMSpector.app*

*(If you don’t know the path, just drag the LCMSpector app into the Terminal window after typing the command `*xattr -d com.apple.quarantine ` *to auto-fill the path, then press Enter).*

1. LCMSpector should now open.

**Linux Users (Docker Run)**

1. Make sure Docker is installed.
2. Pull the image:

docker pull mateuszfido/lcmspector:latest

1. Run the container with display access (adjust your OS syntax if needed):
2. xhost +local:docker
3. docker run -it \
4. -e DISPLAY=$DISPLAY \
5. -v /tmp/.X11-unix:/tmp/.X11-unix \
6. -v /path/to/data_folder:/data \ mateuszfido/lcmspector:latest

*(If you’re not confident with these steps, let us know — we can give you a ready-to-run Docker command for your OS.)*

**Part 2: Getting the Sample Dataset**

1. Go to the link: <https://polybox.ethz.ch/index.php/s/zZsKCd3jGEJdK9c>
2. Click the download button (usually says **Download ZIP**).
3. Once downloaded, **right-click** the .zip file → **Extract All** (Windows) or **Doubleclick** (macOS) to unzip.
4. This will create a folder with one or more .mzML or .txt files. **Do not rename the files.**

**Part 3: Opening the Data in LCMSpector**

1. Open LCMSpector from your Applications or Start Menu. You will see an empty main window.
2. **Drag and drop** one of the sample .mzML or .txt files into the appropriate window in LCMSpector. It’s also possible to drag and drop an entire folder. LCMSpector will automatically recognize the appropriate files and pass them through. *(You can also use the* ***Browse*** *button)*
3. The file name should now appear in the data list area.

**Part 4: Choosing an Ion (Compound) List**

1. Look for the **Ion List** in the “Ion List” panel on the right.
2. To use a **preset list**:
   - Click the dropdown menu
   - Choose one from the available built-in presets (e.g., “Amino acids”).
3. To **create your own list**:
   - Click **New List** or **Edit List**.
   - Type the names or identifiers you want to search for and their m/z values.

**Part 5: Processing the Data**

1. Click the big **Process** button.
2. You may notice a progress bar or activity indicator — processing times vary depending on file size.
3. Once complete, LCMSpector will switch to a results view automatically.

**Part 6: Exploring the Results**

You should now see several panels/plots you can click or switch between:

- **Raw Chromatogram View** – the detector signal from your chromatography analysis, allowing you to click on any time point and showing the underlying mass spectrum in the Raw MS View
- **Raw MS View –** the MS signal updating each time you click a point on the chromatogram above
- **Annotated Chromatogram View** – the peaks for your compounds annotated with the MS signals
- **TIC/XIC time traces** – total or extracted ion traces for every m/z value you selected **Tips for exploring:**
- Hover over peaks to see their retention times and intensities.
- Zoom in by clicking and dragging over an area.
- Right-click on plots to play with the plotting options or export the figures/data from the plot.
- If available, try enabling/disabling overlays to declutter the view.

**Part 7: Exporting Your Results**

Once you’ve explored the results:

**Option 1 – Keyboard Shortcut**

- Press **Ctrl + E** (Windows/Linux) or **Cmd + E** (macOS).

**Option 2 – Menu**

1. Go to the **File** menu.
2. Select **Export**.

This will create a .csv file with the values and concentrations for all the compounds you traced.

You can also save data or graphs directly from the “Results” tab plots in:

- PNG, JPG, SVG (images)
- CSV (data tables)

Choose a save location you can easily find (e.g., Desktop or Documents) and note the file name.

**Part 8: Test Complete**

At this point, you have:

1. Installed LCMSpector
2. Opened sample data
3. Chosen an ion list
4. Run the analysis
5. Explored interactive plots
6. Exported results

You can now complete the [**Usability Test Form** (https://forms.gle/X1cVyH85fXoZHekr9)](https://forms.gle/X1cVyH85fXoZHekr9) and submit it. Thank you!

# Appendix B

## Contents of the usability study questionnaire

1. Device/operating system (Windows/MacOS/Linux?)

___________

1. LCMSpector version (e.g., 0.9.0-fix, 0.9.1, etc.)

___________

1. Was the installation process straightforward?

- Yes
- No

1. Any warnings/pop-ups/problems?

___________

1. Rate the difficulty of installing

- 1 – very easy
- 2
- 3
- 4
- 5
- 6 – impossible

1. Was it clear how to load a file?

- Yes
- No

1. Did the drag&drop work?

- Yes
- No
- Didn’t use it

1. Was it clear when the file was loaded?

- Yes
- No

1. Was it clear how to choose an ion list?

- Yes
- No

1. How would you rate the availability of compounds you were interested in?

- 1
- 2
- 3
- 4
- 5

1. Was starting the processing step clear?

- Yes
- No

1. Could you tell the processing was working?

- Yes
- No

1. Easy to switch views?

- Yes
- No

1. Was exporting the results straightforward?

- Yes
- No

1. Was it clear where the results are saved?

- Yes
- No

1. How clear was it to use the plot export menu?

- 1 -- very easy
- 2
- 3
- 4
- 5 -- very confusing

1. Overall, how would you rate the ease of use?

- 1
- 2
- 3
- 4
- 5
- 6

1. Points where you felt lost

___________

1. What did you like the most about the software?

___________

1. What do you think would make it easier for a newcomer?

___________
