# Supplementary material for: LCMSpector: A simple open-source viewer for targeted hyphenated mass spectrometry analysis
Source: PLoS Comput Biol. 2025 Dec 15;21(12):e1013095. doi: 10.1371/journal.pcbi.1013095 (PMC12725707; doi:10.1371/journal.pcbi.1013095)
Supplement: Attachment — Submitted filename: Reviewer report.docx [file pcbi.1013095.s002.docx]

Manuscript PCOMPBIOL-D-25-00816

*LC-Inspector: A Simple Open-Source Viewer for Targeted Hyphenated Mass Spectrometry Analysis*

Review summary
The authors present a novel and innovative solution for addressing the challenges in mass spectrometry data analysis, particularly for non-experts. The software simplifies the processing and interpretation of complex mass spectrometry data, making it accessible to a wider audience. The open-source nature and the fact that it operates entirely locally are distinct advantages that set it apart from proprietary, server-based alternatives. They also demonstrate utility across diverse MS data (LC/GC-MS, SESI-MS)

With the growing importance of mass spectrometry in fields such as metabolomics, proteomics, and clinical diagnostics, the software meets a critical need for efficient data processing and analysis tools. Its versatility in handling data from multiple vendors and instruments significantly broadens its utility.
The authors presented a well-detailed methodology section, particularly on the preprocessing steps such as baseline correction (using SNIP) and real-time data visualization. The use of PyQt6 for the graphical user interface and pyqtgraph for dynamic data processing ensures that the tool is both efficient and user-friendly.
Additionally, the authors provide robust evidence for the utility and applicability of LC-Inspector by demonstrating its use with publicly available datasets from multiple instruments and vendors. The reproducibility of results across different datasets reinforces its potential for broad adoption in the research community.

Area of concerns

1. While the authors included detailed descriptions of the technical aspects of the software (e.g., SNIP algorithm for baseline correction), the explanation may be too complex for non-specialists. Some of the mathematical details, such as those on the SNIP algorithm, could be further simplified or explained with additional context.

Limited distinction from existing tools (e.g., MZmine, XCMS, ProteoWizard). The authors claim uniqueness in simplicity but fails to benchmark against comparable tools.

No novel algorithms: The baseline correction uses established SNIP (Statistical Non-linear Iterative Peak clipping), identical to the hplc-py package

Suggestion: Consider adding a more intuitive description of the algorithms and their application. A brief overview or a flowchart explaining the data flow within LC-Inspector could enhance understanding for less technical audiences.

2. Although the manuscript demonstrates LC-Inspector versatility with multiple datasets, it lacks a detailed quantitative evaluation of the software performance, especially in terms of speed, accuracy, and memory usage when handling large datasets.

No performance metrics: Processing speed, memory usage, or scalability tests are absent (e.g., handling ‘millions of data points’ claimed in line 89–94 is unsupported).

Reproducibility gaps: Installation dependencies (Python libraries, Qt bindings) are unspecified. Version control is incomplete (GitHub link provided, but no commit hash/version for review).

Validation insufficiency: Applied datasets (line180–188) lack ground-truth comparisons. For example, SESI-MS results (Fig. 3) are not validated against established pipelines.

No case studies showing impact on biological discovery. Examples are limited to technical demonstrations without biological context.

Quantitative features (line 161–170) lack validation against gold-standard methods (e.g., no correlation statistics for concentration calculations).

Suggestion: Include benchmarks comparing LC-Inspector performance against existing tools in terms of processing speed and computational efficiency. Also provide validation against gold-standard methods (e.g., no correlation statistics for concentration calculations This would provide concrete evidence of its advantages and limitations.

3. The authors does not discuss potential limitations of the software. For instance, how does LC-Inspector handle very large datasets or data from instruments with different calibration schemes?

Suggestion: A section discussing the known limitations of LC-Inspector would be beneficial. Specifically, limitations in scalability, data handling, or compatibility with other mass spectrometry workflows should be addressed.

4. The manuscript briefly touches upon the graphical user interface (GUI) design, but more detailed discussion of its usability is needed. How intuitive is the interface for a non-expert? Are there tutorials or documentation available to guide new users?

Suggestion: Expand on the user experience aspect by providing feedback from actual users, or by including results from a usability study. Additionally, providing access to user manuals or a demonstration video could further enhance the tool's appeal to non-experts.

5. While LC-Inspector supports mzML, TXT, and CSV formats, the manuscript does not explore the possibility of supporting other popular formats in the mass spectrometry community, such as mzXML or the proprietary formats from specific instrument vendors.

Suggestion: Consider expanding the supported input formats to include more proprietary formats and provide guidance on how users can contribute or extend format support. Additionally, offering a plugin architecture could allow users to integrate LC-Inspector with other analysis tools.

6. The authors mentions the availability of the source code on GitHub, but it does not emphasize the importance of version control and community contributions to the software’s development.

Suggestion: Explicitly mention the version control system used (e.g., Git) and encourage contributions from the community. A roadmap for future development and potential collaborations would also be useful.

Minor issues

1.Baseline Correction (Page 7, Lines 135-150): The use of the SNIP algorithm for baseline correction is well-described, but the mathematical formulation could be more accessible to readers unfamiliar with the specific algorithm.

2.Software Functionality (Page 5, Lines 103-113): The authors explained how the user can interact with the interface, but it lacks detailed descriptions of specific features like error handling or how users can manage unexpected results (e.g., when an input file is corrupt).

Minor Revision

Figures

Figure 1. Clarify panel labels (e.g., "architecture" in 1A is vague).

Fig. 2. Specify preprocessing parameters for SNIP.

Main text

Define ‘high-complexity data’ (line 36) quantitatively (e.g., sample size, dimensionality).

Cite literature for SNIP's efficacy in MS contexts (line136–149).

Freeze code version (e.g., Zenodo DOI for exact version reviewed) and add unit tests to the codebase.

Conclusion

This manuscript present LC-Inspector as an innovative tool that addresses a significant gap in the field of mass spectrometry data analysis, particularly for non-specialists. The manuscript demonstrates the software's capabilities and provides a clear explanation of its functionality. However, further improvements are needed in terms of performance benchmarking, a more detailed evaluation of its limitations, and usability for non-experts.
