# Supplementary material for: LCMSpector: A simple open-source viewer for targeted hyphenated mass spectrometry analysis
Source: PLoS Comput Biol. 2025 Dec 15;21(12):e1013095. doi: 10.1371/journal.pcbi.1013095 (PMC12725707; doi:10.1371/journal.pcbi.1013095)
Supplement: Attachment — Submitted filename: Response to Reviewers.docx [file pcbi.1013095.s005.docx]

Manuscript PCOMPBIOL-D-25-00816

Response to Reviewers

Dear Prof. Taghizadeh and Prof. Zhang,

Enclosed you will find the revised manuscript “LC-Inspector: a simple open-source viewer for targeted hyphenated mass spectrometry analysis” for publication in PLOS Computational Biology.

Thank you very much for the opportunity to respond to the comments on our manuscript. We carefully considered the comments and revised the manuscript accordingly. We appreciate the reviewers’ feedback and have addressed all the points below. The reviewers’ comments are written in blue for clarity.

We believe the revised manuscript is now stronger and much more complete. We look forward to your response.

**Points raised by Reviewer 1**

Reviewer #1: The authors did a great job on this manuscript LC-Inspector: a simple open-source viewer for targeted hyphenated mass spectrometry analysis to PLOS Computational Biology. The development of LC-Inspector is a commendable contribution to the mass spectrometry community, particularly in providing an accessible, user-friendly, and open-source tool for non-experts in the field. I have carefully reviewed your manuscript and would like to provide some constructive feedback to help improve its clarity and overall impact.

Area of concerns

1. While the authors included detailed descriptions of the technical aspects of the software (e.g., SNIP algorithm for baseline correction), the explanation may be too complex for non-specialists. Some of the mathematical details, such as those on the SNIP algorithm, could be further simplified or explained with additional context.

Limited distinction from existing tools (e.g., MZmine, XCMS, ProteoWizard). The authors claim uniqueness in simplicity but fails to benchmark against comparable tools.

No novel algorithms: The baseline correction uses established SNIP (Statistical Non-linear Iterative Peak clipping), identical to the hplc-py package

Suggestion: Consider adding a more intuitive description of the algorithms and their application. A brief overview or a flowchart explaining the data flow within LC-Inspector could enhance understanding for less technical audiences.

Thank you for this remark.

Following this suggestion, we deconstructed the explanation of the SNIP algorithm into a series of steps, providing reasoning for each step with the aim to simplify and provide more detail on how it is applied to chromatography data within our software (p7:188).

In terms of algorithm novelty, we expanded upon the explanation of how LCMSpector parses text-based input and traces the ions we use for analysis, both of which, to our knowledge, constitute original algorithms.

Following this advice, we also replaced the schematic explaining the overall data flow of the application and placed the model-view-controller design figure in the Supporting Information (Figure 1 of main text, Supporting Information Figure S1).

2. Although the manuscript demonstrates LC-Inspector versatility with multiple datasets, it lacks a detailed quantitative evaluation of the software performance, especially in terms of speed, accuracy, and memory usage when handling large datasets.

No performance metrics: Processing speed, memory usage, or scalability tests are absent (e.g., handling ‘millions of data points’ claimed in line 89–94 is unsupported).

Reproducibility gaps: Installation dependencies (Python libraries, Qt bindings) are unspecified. Version control is incomplete (GitHub link provided, but no commit hash/version for review).

Validation insufficiency: Applied datasets (line180–188) lack ground-truth comparisons. For example, SESI-MS results (Fig. 3) are not validated against established pipelines.

No case studies showing impact on biological discovery. Examples are limited to technical demonstrations without biological context.

Quantitative features (line 161–170) lack validation against gold-standard methods (e.g., no correlation statistics for concentration calculations).

Suggestion: Include benchmarks comparing LC-Inspector performance against existing tools in terms of processing speed and computational efficiency. Also provide validation against gold-standard methods (e.g., no correlation statistics for concentration calculations This would provide concrete evidence of its advantages and limitations.

Thank you very much for this suggestion. Following this advice, we conducted thorough performance and scalability tests on all platforms for which LCMSpector is currently distributed, which is Windows, macOS, and Linux. We tested different numbers of mass spectrometry files and measured loading time and memory usage on every platform and compared them to the latest versions of two established open-source alternatives with very similar functionality: mzio’s MZMine and OpenMS TOPPView (new, Figure 5C-E).

We also validated LCMSpector’s quantitative capabilities by comparing it to what can be considered a “golden standard”, which is the vendor’s proprietary quantification algorithm (ThermoFisher Scientific’s LC-MS software suite Chromeleon). By measuring the intensities of 5 standard compounds (amino acid solutions), we tested which program produces the most precise and accurate quantification results. We created calibration curves from the integrated intensities reported by each software’s integration algorithm and tested the accuracy with which it would predict one of the concentrations interpolated from the calibration curve (new, Figure 5A-B and SI Figures S4-S18).

We also performed a case study on an already published dataset (Fig. 3) in order to demonstrate the biological significance of the application’s functionality. We found several peaks which contribute to the total abundance of compounds analyzed by the authors, which were not included in their quantification workflow and which can significantly influence the outcome of the analysis.

With regards to the dependencies, none are required for the installation of executables versions of LCMSpector, however for the usage of command-line interface versions the dependencies are listed in the README file of the GitHub repository and the requirements.txt file – we now also mention this in the main text (p22:545). Although LCMSpector is under constant development, the version for review should be the same as used for the benchmarking (release 0.9.10, commit hash 95ad0e1, available on GitHub at <https://github.com/MateuszFido/LCMSpector/releases/tag/v0.9.10>), so that consistency with the manuscript content is preserved. We now also provide this information in the main text (p21:533).

3. The authors does not discuss potential limitations of the software. For instance, how does LC-Inspector handle very large datasets or data from instruments with different calibration schemes?

Suggestion: A section discussing the known limitations of LC-Inspector would be beneficial. Specifically, limitations in scalability, data handling, or compatibility with other mass spectrometry workflows should be addressed.

Thank you for this point. In the section “Availability and future directions” we now included a description of the current limitations of LCMSpector. We also address these points in the newly created roadmap (Figure S2, Supporting Information).

4. The manuscript briefly touches upon the graphical user interface (GUI) design, but more detailed discussion of its usability is needed. How intuitive is the interface for a non-expert? Are there tutorials or documentation available to guide new users?

Suggestion: Expand on the user experience aspect by providing feedback from actual users, or by including results from a usability study. Additionally, providing access to user manuals or a demonstration video could further enhance the tool's appeal to non-experts.

Thank you for these questions. Following this advice, we expanded the available installation, usage and learning resources for LC-Inspector to make its adoption as seamless as possible for new users. This includes documentation in form of a wiki-style section of the GitHub repository, to which we now link in the main manuscript (p22:549). The wiki contains detailed usage and installation instructions, with short videos and graphical demonstrations of how data can be uploaded, processed, plotted, and exported from LCMSpector.

5. While LC-Inspector supports mzML, TXT, and CSV formats, the manuscript does not explore the possibility of supporting other popular formats in the mass spectrometry community, such as mzXML or the proprietary formats from specific instrument vendors.

Suggestion: Consider expanding the supported input formats to include more proprietary formats and provide guidance on how users can contribute or extend format support. Additionally, offering a plugin architecture could allow users to integrate LC-Inspector with other analysis tools.

Thank you for this suggestion.

Supporting other open mass spectrometry formats is one of the first goals of LCMSpector’s future development – we now mention this in the roadmap attached in the Supporting Information (Fig. 2 Supporting Information). Additionally, LCMSpector currently implements text parsing for chromatography/ion mobility data and implementing text-based mass spectrometry formats would be a relatively straightforward next step.

Incorporating vendor formats is one of the biggest challenges LCMSpector and virtually all mass spectrometry-adjacent software struggle with. The main reason for this is that these formats are kept in binary format, unreadable without the use of vendor-developed readers which depend on .NET and C# libraries and use legacy APIs functioning exclusively under Windows. The main workaround would be to statically link either MSConvert (ProteoWizard’s conversion utility) or the readers themselves, and this is what, e.g., mzio’s MZMine does – but this functionality unfortunately only works on Windows. The file formats themselves often undergo change as new functionality is added or modified by the vendor. Nonetheless, we have expanded on this now in the “Availability and future directions” section, as well as the roadmap for future development.

6. The authors mentions the availability of the source code on GitHub, but it does not emphasize the importance of version control and community contributions to the software’s development.

Suggestion: Explicitly mention the version control system used (e.g., Git) and encourage contributions from the community. A roadmap for future development and potential collaborations would also be useful.

Thank you for this suggestion. We now explicitly mention the version control system and added a roadmap (Figure S2, Supporting Information). On LCMSpector’s GitHub page we have also added contribution instructions, an issue creation template and Docker installation instructions for a unified development environment.

Minor issues

1.Baseline Correction (Page 7, Lines 135-150): The use of the SNIP algorithm for baseline correction is well-described, but the mathematical formulation could be more accessible to readers unfamiliar with the specific algorithm.

Thank you for this comment. We now corrected the description of the SNIP algorithm, as mentioned above (starting p7:188).

2.Software Functionality (Page 5, Lines 103-113): The authors explained how the user can interact with the interface, but it lacks detailed descriptions of specific features like error handling or how users can manage unexpected results (e.g., when an input file is corrupt).

Thank you for this comment, we expanded the functionality description to explain how errors are logged and handled, especially in the preprocessing segment (p5:154-160).

Minor Revision

Figures

Figure 1. Clarify panel labels (e.g., "architecture" in 1A is vague).

Thank you, the label was corrected to explain the panel in more detail.

Fig. 2. Specify preprocessing parameters for SNIP.

Thank you, preprocessing parameters were added to the figure description.

Main text

Define ‘high-complexity data’ (line 36) quantitatively (e.g., sample size, dimensionality).

Thank you, we were referring to the dimensionality of the data in this statement, not necessarily the sample size, and updated accordingly.

Cite literature for SNIP's efficacy in MS contexts (line136–149).

In LCMSpector we only use SNIP for chromatographic signals – MS signal baseline correction is done using a simpler algorithm performing linear baseline approximation and subtraction. We have now clarified it in the MS processing section (p9:241).

Freeze code version (e.g., Zenodo DOI for exact version reviewed) and add unit tests to the codebase.

Thank you for this suggestion. Zenodo DOI, Github commit hash for review are available in the section “Availability and future directions”. Unit and integration tests have also been added to the codebase, increasing overall test coverage to about 90%.

Conclusion

This manuscript present LC-Inspector as an innovative tool that addresses a significant gap in the field of mass spectrometry data analysis, particularly for non-specialists. The manuscript demonstrates the software's capabilities and provides a clear explanation of its functionality. However, further improvements are needed in terms of performance benchmarking, a more detailed evaluation of its limitations, and usability for non-experts.

Thank you again for these points. We believe the changes, inclusions of performance metric assessments as compared to other viable open-source tools, an overview of its limitations and a clearer explanation of usability for non-experts have improved our manuscript substantially.

**Points raised by Reviewer 2**

Reviewer #2: Fido et al. present LC-Inspector, which seems to be a useful utility for hyphenated MS data analysis. The manuscript is fairly straightforward in describing the software and its capabilities. I think the work is sound and I have no major critique. My rather minor comments below:

p2:32 (and elsewhere)

The abbreviation MS has already been introduced, so use it consistently instead of ‘mass spectrometry’.

Thank you for this comment – “mass spectrometry” has been replaced with “MS” wherever appropriate to reduce repeated usage of the term “mass spectrometry”.

p2:39

“vendor’s” should probably be “vendors’” because plural genitive.

Thank you for this remark, corrected accordingly (p2:43).

p3:55

“LC-Inspector is a completely free-of-charge…” Note that LC-Inspector has not been introduced yet. Consider “Here, we present LC-Inspector, which is a completely free-of-charge…”

Thank you for this remark, corrected accordingly (p3:71).

p3:64

“...concurrency and futures modules.” I suspect the authors mean “…concurrency.future modules”. Unless there coincidentally is a separate futures module that I cannot find.

Thank you for this remark, we clarified this point (p3:80).

p3:72-47

Text or csv files can have their data stored in many ways. If certain column orders or similar are expected, please mention that.

Thank you for this remark, we have now clarified the text format parsing (p3:90).

p4:85

“It then rebuilds the extracted ion chromatograms for those m/z ranges…” Maybe it is a convention I am unfamiliar with, or I misunderstand something, but is the term ‘ion chromatogram’ used for ions in a mass spectrometer? If so, fine, but if not, I suggest referring to it by another name, like a reduced mass spectrum or something. Especially since chromatography data is also part of the context. If I misunderstand, at least consider that the text can be misinterpreted as it is currently written.

Thank you for this comment – the term “ion chromatogram” is indeed often used in mass spectrometry meaning ion intensity (detector’s response) as a function of time. XIC/EIC (extracted ion chromatogram) has become a common name for a graph displaying a time trace of intensity for a small range of m/z values, usually corresponding to a peak of interest, “extracted” from the full mass spectrum. This is usually meant in contrast to the TIC (total ion current/chromatogram), displaying the total intensity of all ions in the sample over its run time.

For clarity, we changed the term to “time-dependent ion traces (extracted ion chromatograms, XICs)” (p4:117).

p4:96

“comma-separated values (CSV)” CSV has already been discussed, so no point in introducing the abbreviation here.

Thank you, the abbreviation was removed (p4:128).

p7:145

It would be prudent to cite the original paper Hampton et al. (https://doi.org/10.1016/0168-9002(94)91657-8) in relation to the LLS operator.

Thank you for this remark, we included the appropriate citation.

Lastly, I am curious about the name of the software. LC suggests liquid chromatography, but the central technique here is MS. Is there some history behind the name?

The main goal of naming the software was to combine the words LC and MS with the concept of “inspection” or “investigation”. Only afterwards did we conclude that the software can in principle be used with any mass spectrometry technique as well as other chromatography techniques (GC/IC/SEC/etc.) on their own, or hyphenated to MS.

However, we realize that not putting “MS” in the title will likely steer the potential user towards thinking that the software might be focused on liquid chromatography, which is not the case. Therefore, we chose to rename the software to “LCMSpector”, to include the “MS” abbreviation while still maintaining the original naming convention.
