# Supplementary material for: LCMSpector: A simple open-source viewer for targeted hyphenated mass spectrometry analysis
Source: PLoS Comput Biol. 2025 Dec 15;21(12):e1013095. doi: 10.1371/journal.pcbi.1013095 (PMC12725707; doi:10.1371/journal.pcbi.1013095)
Supplement: Attachment — Submitted filename: Response_to_Reviewers_auresp_2.docx [file pcbi.1013095.s007.docx]

Manuscript PCOMPBIOL-D-25-00816

Response to Reviewers

Dear Prof. Taghizadeh and Prof. Zhang,

Thank you for the opportunity to improve our manuscript. Enclosed you will find the second revision of our manuscript “LCMSpector: a simple open-source viewer for targeted hyphenated mass spectrometry analysis” for publication in PLOS Computational Biology. We carefully considered the comments and revised the manuscript accordingly. We appreciate the reviewers’ feedback and have addressed all the points below. The reviewers’ comments are written in blue for clarity.

**Points raised by Reviewer 1**

Thank you for the opportunity to improve the manuscript. We look forward to your response.

Despite the revisions are excellent, two minor points could be considered
1. The response letter mentions increasing test coverage to ‘about 90%’. For maximum transparency, the exact test coverage percentage could be included in the "Availability" section or the GitHub repository.

Thank you for this comment. As the codebase has expanded since our previous revision, we now added the current test coverage (82%) with a more detailed explanation in the “Availability” section of the manuscript.

2. The stability issues noted in the usability study (crashes during simultaneous operations) are acknowledged as a point for future improvement. While it's good they are noted, a brief sentence in the ‘Limitations’ section could further demonstrate transparency.

Thank you for this point. We have amended the “Limitations” section to include this information.

Additionally, we have amended Fig. 1 and Appendix A to remove any potential copyright issues (buttons and graphical interfaces of the operating system or GitHub website) and formatted our files and figures according to the journal’s requirements. We are happy to have been able to improve our submission and look forward to your response.

Warm regards,

Mateusz Fido, on behalf of all authors
