# Supplementary material for: LCMSpector: A simple open-source viewer for targeted hyphenated mass spectrometry analysis
Source: PLoS Comput Biol. 2025 Dec 15;21(12):e1013095. doi: 10.1371/journal.pcbi.1013095 (PMC12725707; doi:10.1371/journal.pcbi.1013095)
Supplement: Attachment — Submitted filename: Reviewer comment .docx [file pcbi.1013095.s004.docx]

Dear Authors,

I have carefully examined your point-by-point responses and the revised manuscript. I am pleased to note that you have been exceptionally responsive and have undertaken a comprehensive revision that has substantially elevated the quality, rigor, and impact of your work. The additions and clarifications have effectively mitigated the core concerns I have raised in the initial review.

Specifically, the following revisions are particularly noteworthy and have enhanced the manuscript from a promising software description into a convincing and validated resource for the community

1. Introduction of rigorous performance benchmarks, the authors addition of head-to-head performance comparisons with established tools like MZmine and OpenMS TOPPView (Figure 5C-E) is a critical improvement. By quantifying loading times, processing speed, and memory footprint across three operating systems, you have moved beyond qualitative claims of efficiency to provide concrete, empirical evidence. The finding that LCMSpector achieves competitive speed while consuming significantly less memory is a strong and distinct advantage that you have now convincingly demonstrated.

2. Validation of quantitative fidelity against a gold standard, the manuscript quantitative analysis comparing LCMSpector's concentration estimates against those from the vendor's own algorithm (Chromeleon) and MZmine (Figure 5A-B) is exactly the type of validation required to build trust in a new analytical tool. The results, which intriguingly show superior performance of the open-source tools in this specific direct-infusion context, provide a powerful argument for the accuracy and reliability of your software's core algorithms.

3. Demonstration of biological utility through a case study, the authors new analysis of a published liver disease dataset (Figure 3) successfully bridges the gap between technical capability and biological discovery. This no longer feels like a simple demonstration, but an actual research application. The insight regarding the potential impact of dimer peaks on bile acid quantification is a perfect example of how your software's integrated, raw-data-viewing capability can lead to more accurate and insightful analyses a key selling point that is now powerfully substantiated.

4. Empirical support for user-friendliness, conducting a formal usability study (Figure 6) was an excellent initiative that moves discussions of the GUI from anecdotal to evidence-based. The positive feedback from novice users provides robust, independent validation of your central thesis: that LCMSpector successfully lowers the barrier to entry for complex MS data analysis.

5. Enhanced scholarly transparency and reproducibility**,** the authors’ inclusion of a dedicated ‘Limitations’ section, a future development roadmap (Fig. S2), and the freezing of the code version with a specific commit hash and Zenodo DOI represent best practices in open-source software publication. This level of transparency not only builds immediate credibility but also thoughtfully sets community expectations and facilitates future collaboration and development.

6. Improved conceptual and algorithmic clarity, the restructuring of the SNIP algorithm explanation into a step-by-step process and the detailed computational analysis of the XIC construction algorithm greatly enhance the manuscript's educational value and clarify the novel contributions of your implementation, effectively distinguishing it from a mere repackaging of existing libraries.


Despite the revisions are excellent, two minor points could be considered

1. The response letter mentions increasing test coverage to ‘about 90%’. For maximum transparency, the exact test coverage percentage could be included in the "Availability" section or the GitHub repository.

2. The stability issues noted in the usability study (crashes during simultaneous operations) are acknowledged as a point for future improvement. While it's good they are noted, a brief sentence in the ‘Limitations’ section could further demonstrate transparency.

The authors have undertaken a rigorous and highly effective revision process. They have responded to all my comments and significantly enhanced the scientific rigor, clarity, and impact of their work through the addition of extensive new data and analyses. The manuscript now provides convincing evidence for the performance, accuracy, and user-friendliness of LCMSpector, effectively positioning it as a valuable new open-source tool for targeted mass spectrometry analysis.

The revisions have fully addressed all my major and minor concerns. The manuscript now meets the high standards expected for a software publication in PLOS Computational Biology. The manuscript is now ready to proceed further for the Editor-in-Chief’s final decision.
