# Supplementary material for: LCMSpector: A simple open-source viewer for targeted hyphenated mass spectrometry analysis
Source: PLoS Comput Biol. 2025 Dec 15;21(12):e1013095. doi: 10.1371/journal.pcbi.1013095 (PMC12725707; doi:10.1371/journal.pcbi.1013095)
Supplement: Attachment — Submitted filename: Reviewer comment.docx [file pcbi.1013095.s006.docx]

**Reviewer comment**


**LCMSpector: a simple open-source viewer for targeted hyphenated mass spectrometry analysis**

I am pleased with the authors’ careful and thorough responses to my previous comments. The revisions made to the manuscript have fully addressed the two minor issues I raised.

Regarding test coverage, the authors have now included the exact test coverage percentage (82%) in the ‘Availability’ section. This provides maximum transparency as requested and is a welcome addition.

Regarding stability issues, the authors have amended the ‘Limitations’ section to explicitly mention the stability issues encountered by some users during the usability study. This demonstrates a commendable level of transparency about the current state of the software and its areas for future improvement.

The authors have also taken care to address other editorial points, such as figure formatting. The manuscript is now significantly strengthened, clearly written, and presents a valuable open-source tool for the community. I have no further concern. The manuscript is now highly rigorous and scientifically sound which make it fit for publication in PLOS Computational Biology. The work is now ready to go further for the Editor-in-Chief’s final decision in line with the remaining peer reviewers’ comments.
